# Automotive Telemetry in Connected Vehicles: A Review of 5G and Satellite Communication Technologies for Long-Range Data Transmission

**DOI:** 10.3390/s26154968

**Published:** 2026-08-05

**Authors:** Jozef Jaroslav Fekiač, Lucia Kakošová, Michal Krbata, Marcel Kohutiar, Alena Breznická, Pavol Mikuš, Maroš Eckert

**Affiliations:** Faculty of Special Technology, Alexander Dubcek University of Trenčín, 911 06 Trenčín, Slovakia; lucia.kakosova@tnuni.sk (L.K.); michal.krbata@tnuni.sk (M.K.); marcel.kohutiar@tnuni.sk (M.K.); alena.breznicka@tnuni.sk (A.B.); pavol.mikus@tnuni.sk (P.M.); maros.eckert@tnuni.sk (M.E.)

**Keywords:** automotive telemetry, connected vehicles, 5G communication, satellite communication, V2X, internet of vehicles, software defined vehicles, digital twins, intelligent transportation systems, cybersecurity

## Abstract

The rapid evolution of connected and autonomous vehicles has significantly increased the importance of automotive telemetry as a fundamental component of intelligent transportation systems. Modern vehicles continuously generate large volumes of operational, environmental, and behavioral data that must be transmitted, processed, and analyzed in real time to support applications such as predictive maintenance, remote diagnostics, cooperative mobility, and autonomous driving. The growing demand for reliable and large-scale data exchange has highlighted the critical role of advanced communication infrastructures in connected mobility ecosystems. This review provides a comprehensive overview of automotive telemetry technologies with a particular focus on communication solutions enabling long-range data transmission. The study examines the architecture of automotive telemetry systems, connected vehicles, and the Internet of Vehicles, followed by an analysis of Vehicle-to-Everything (V2X) communication, fifth-generation (5G) mobile networks, satellite communication systems, and emerging hybrid 5G–satellite architectures. In addition, the review discusses Software Defined Vehicles, Digital Twin technologies, artificial intelligence integration, and cybersecurity challenges associated with highly connected transportation environments. The analysis demonstrates that no single communication technology can satisfy all requirements of future intelligent mobility systems. Instead, hybrid communication architectures combining terrestrial and non-terrestrial networks are expected to provide the reliability, coverage, scalability, and low-latency performance required for next-generation connected transportation. Finally, key research challenges and future development directions are identified, emphasizing the growing convergence of telemetry, communication networks, artificial intelligence, digital twins, and cybersecurity within future connected mobility ecosystems.

## 1. Introduction

The automotive industry is experiencing a rapid transformation driven by advances in digital technologies, communication systems, artificial intelligence, and automation. Modern vehicles have evolved from purely mechanical transportation systems into highly interconnected cyber-physical platforms capable of generating, processing, and exchanging vast amounts of information in real time. This transformation has led to the emergence of connected and autonomous vehicles, which are increasingly considered fundamental building blocks of intelligent transportation systems. Abdelkader et al. [1] described connected vehicles as a key enabling technology for future mobility, while Ahmed et al. [2] highlighted their potential to improve transportation efficiency, road safety, and traffic management. Similarly, Pipicelli et al. [3] emphasized that connected and autonomous vehicles represent one of the most important technological directions in the evolution of modern transportation.

A critical component supporting this transformation is automotive telemetry. Telemetry systems enable the continuous acquisition, transmission, and analysis of vehicle data collected from numerous onboard sensors, electronic control units, positioning systems, and monitoring devices. Such systems provide information related to vehicle dynamics, powertrain operation, battery condition, tire performance, driver behavior, and environmental conditions. The collected data can be utilized for predictive maintenance, fleet management, remote diagnostics, safety monitoring, and autonomous driving applications. Recent studies have demonstrated the growing importance of telemetry in intelligent transportation environments. Galinski et al. [4] introduced a comprehensive dataset combining camera and telemetry information collected from real road environments under varying weather and lighting conditions. De Lima et al. [5] demonstrated the applicability of telemetry-derived data for driver behavior analysis and classification, while Nasreddine et al. [6] highlighted the increasing importance of advanced communication infrastructures for supporting next-generation automotive services.

The continuous growth in vehicle-generated data has created significant demands on communication networks. Traditional communication infrastructures often struggle to provide the low latency, high reliability, scalability, and ubiquitous coverage required by modern connected vehicle applications. These requirements become particularly important in scenarios involving real-time vehicle monitoring, autonomous driving, cooperative mobility, and large-scale fleet management. Consequently, the development of advanced communication technologies has become one of the key research priorities in the field of intelligent transportation systems.

Among the available communication technologies, fifth-generation (5G) mobile networks have emerged as one of the most promising solutions for automotive telemetry applications. The capabilities of 5G networks, including enhanced mobile broadband, ultra-reliable low-latency communication, and support for massive machine-type communications, enable efficient handling of large volumes of vehicle-generated data. Gohar and Nencioni [7] highlighted the importance of 5G technologies for intelligent transportation systems, while Condoluci et al. [8] proposed a system-level architecture supporting 5G-enabled V2X communication. Furthermore, Biswas and Wang [9] demonstrated that the integration of 5G communication, edge intelligence, Internet of Things technologies, and blockchain frameworks can significantly enhance the functionality of connected and autonomous vehicles.

Despite the substantial advantages of terrestrial communication infrastructures, maintaining reliable connectivity remains challenging in rural regions, remote transportation corridors, maritime routes, and other areas with limited cellular coverage. Satellite communication technologies have therefore attracted considerable attention as a complementary solution capable of providing near-global coverage and supporting long-range data transmission. Recent developments in satellite communication systems, non-terrestrial networks, and integrated 5G–satellite architectures have created new opportunities for automotive applications. Kang et al. [10] reviewed current security challenges in satellite communication systems, while Völk et al. [11] investigated the integration of satellite communication within 5G infrastructures. Similarly, Uko et al. [12] demonstrated the potential of integrated 5G–satellite networks for supporting large-scale Internet of Things applications and long-range connectivity.

In addition to communication infrastructure, vehicle-to-everything (V2X) communication has become a fundamental component of connected mobility. V2X technologies enable communication between vehicles, roadside infrastructure, cloud platforms, pedestrians, and other traffic participants, thereby improving situational awareness, traffic efficiency, and road safety. Khalid et al. [13] proposed intelligent approaches for optimizing hybrid V2X communication technologies, whereas Jung et al. [14] experimentally validated the effectiveness of V2X-assisted autonomous driving systems. These developments indicate that future transportation systems will increasingly rely on cooperative communication and data sharing among multiple entities.

The future evolution of automotive telemetry is closely associated with emerging concepts such as Software Defined Vehicles (SDVs), over-the-air software updates, artificial intelligence, and digital twins. These technologies enable continuous software-driven vehicle development, advanced diagnostics, virtual testing, predictive maintenance, and intelligent decision-making. Kim et al. [15] proposed secure mechanisms for over-the-air software updates, while Kaya et al. [16] introduced a comprehensive digital twin framework integrating vehicles, drivers, and road infrastructure within connected mobility environments.

**Figure 1:**
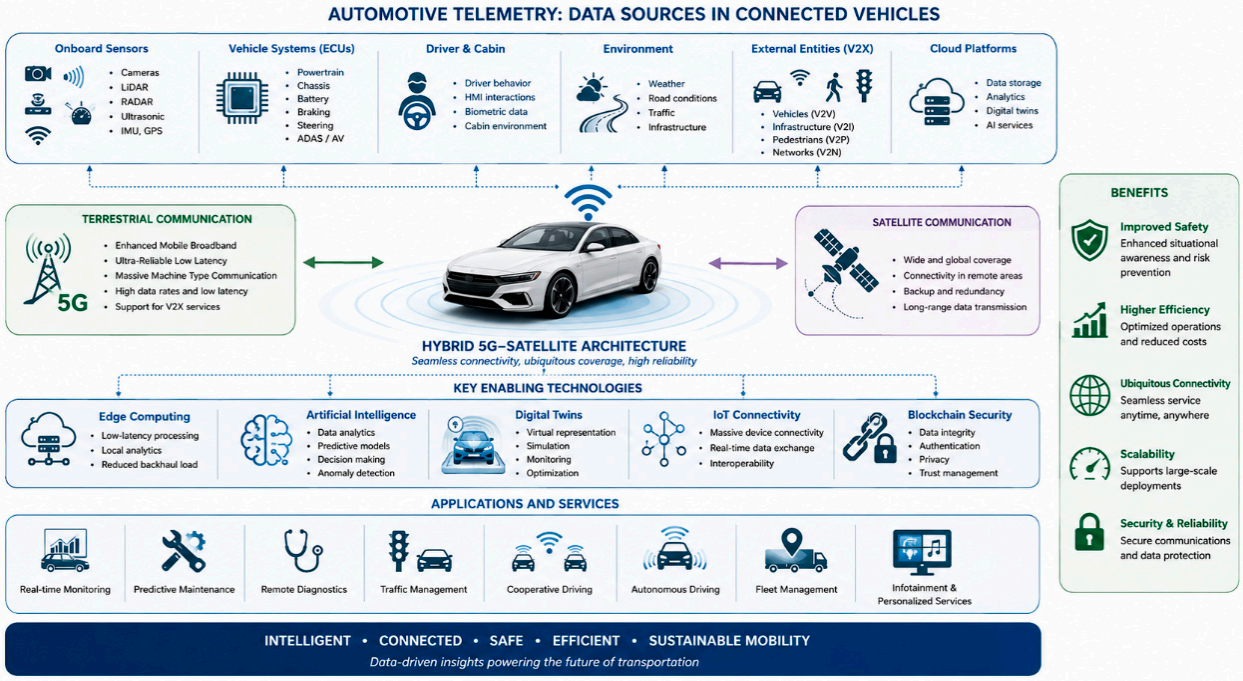
Integrated ecosystem of connected vehicles, communication technologies, and intelligent mobility services.

This review article provides a comprehensive overview of automotive telemetry in connected vehicle ecosystems, with particular emphasis on communication technologies enabling long-range data transmission. The paper analyzes the role of 5G and satellite communication systems, discusses V2X communication architectures and applications, examines cybersecurity challenges, and reviews emerging concepts such as Software Defined Vehicles and Digital Twins. Finally, current research challenges and future development trends are identified to support the continued evolution of intelligent connected transportation systems. Figure 1 provides an overview of the technological ecosystem supporting connected and autonomous vehicles. It illustrates the relationships between automotive telemetry, hybrid 5G–satellite communication, V2X connectivity, artificial intelligence, digital twins, IoT technologies, and cybersecurity mechanisms. Together, these components enable the development of intelligent and data-driven mobility services. Unlike previous surveys, which typically address individual technological domains—such as 5G-enabled intelligent transportation systems, non-terrestrial networks, V2X communication, or vehicular cybersecurity—largely in isolation, this review examines these technologies specifically from the perspective of long-range automotive telemetry and analyzes how their convergence enables reliable, large-scale transmission of vehicle-generated data. To clearly position this work with respect to the existing literature, Table 1 compares its scope with representative recent surveys. The comparison shows that, although each individual domain has been reviewed before, the joint treatment of telemetry architectures, hybrid terrestrial/non-terrestrial communication, V2X, Software-Defined Vehicles, Digital Twins, and cybersecurity within a single telemetry-oriented analytical framework has not been provided by previous review articles.

Beyond this integrative scope, the present review makes four specific contributions. First, it organizes the reviewed domains into a telemetry-oriented taxonomy that explicitly maps enabling communication technologies, intelligence and virtualization layers, and cross-cutting security mechanisms onto the requirements of long-range transmission of vehicle-generated data. Second, it provides quantitative cross-technology comparisons—covering latency, throughput, coverage, scalability, energy efficiency, deployment complexity, and standardization maturity—rather than purely qualitative feature lists. Third, each thematic section is accompanied by a critical synthesis that contrasts competing approaches, highlights conflicting experimental findings, and identifies methodological limitations of the underlying studies. Fourth, the review concludes with a prioritized gap analysis and research roadmap that translates the identified open problems into concrete, time-phased research recommendations.

### Review Methodology

The literature analyzed in this review was identified through systematic searches of the Scopus, Web of Science, and IEEE Xplore databases, complemented by the MDPI and SpringerLink full-text collections. The searches combined the terms “automotive telemetry”, “connected vehicles”, “Internet of Vehicles”, “V2X”, “5G”, “non-terrestrial networks”, “satellite communication”, “software-defined vehicle”, “digital twin”, and “vehicular cybersecurity” in the title, abstract, and keyword fields. The searches primarily covered publications from 2015 onwards, with emphasis on work published within the last five years so that the review reflects the current state of 5G-Advanced and NTN standardization; selected earlier publications were retained where they define fundamental concepts. Records were screened for relevance to the long-range transmission of vehicle-generated data: peer-reviewed journal articles, conference papers, and relevant standardization documents published in English were included, whereas studies addressing purely short-range in-vehicle communication, telemetry in non-automotive domains, or lacking a communication or data-transmission component were excluded. After the removal of duplicates and the screening of titles, abstracts, and full texts, more than one hundred publications were retained and organized according to the taxonomy presented at the end of this review.

## 2. Automotive Telemetry and Connected Vehicle Ecosystem

The rapid advancement of digital technologies has fundamentally transformed the automotive industry over the past two decades. Modern vehicles are evolving from conventional mechanical transportation devices into highly interconnected cyber-physical systems capable of continuously generating, processing, and exchanging large volumes of information. This transformation has been driven by the integration of advanced sensors, electronic control units, communication modules, cloud computing platforms, and intelligent software systems, enabling vehicles to become active components of broader digital mobility ecosystems.

A key technology supporting this evolution is automotive telemetry, which enables the acquisition, transmission, storage, and analysis of vehicle-generated data. Telemetry systems facilitate continuous monitoring of vehicle performance, operational conditions, driver behavior, and environmental influences, supporting applications such as remote diagnostics, predictive maintenance, fleet management, safety monitoring, and autonomous driving.

The growing availability of wireless communication technologies has significantly expanded telemetry capabilities by enabling real-time data exchange between vehicles, cloud platforms, transportation infrastructure, and service providers. As a result, modern transportation systems increasingly rely on data-driven decision-making and cooperative information sharing.

This development has accelerated the emergence of connected vehicles and the Internet of Vehicles (IoV). Connected vehicles communicate not only with external communication networks but also with surrounding vehicles, roadside infrastructure, cloud services, and intelligent transportation platforms. Equipped with advanced sensing technologies, including cameras, radar, LiDAR, positioning systems, and onboard diagnostic units, vehicles continuously generate large-scale datasets describing both their operational status and surrounding environment. These datasets form the foundation for intelligent transportation services, autonomous driving, predictive analytics, adaptive traffic management, and digital twin technologies.

However, the increasing volume of vehicle-generated data also introduces significant challenges related to communication capacity, interoperability, cybersecurity, and real-time data processing. Addressing these challenges is essential for the successful deployment of future connected mobility systems.

Therefore, this chapter provides an overview of automotive telemetry architectures, connected vehicle ecosystems, and the primary sources and categories of data generated within contemporary transportation environments.

### 2.1. Automotive Telemetry Architecture

Automotive telemetry represents a technological framework that enables the acquisition, transmission, processing, and analysis of vehicle-generated data during operation. Initially developed for motorsport and specialized engineering applications, telemetry systems have gradually evolved into essential components of modern connected vehicles. Early implementations primarily focused on wireless transmission of operational parameters from mechanical systems, enabling remote monitoring of component performance and operating conditions. As communication technologies advanced, telemetry systems expanded beyond simple measurement transmission and became integral elements of intelligent transportation ecosystems [20,21].

Contemporary automotive telemetry architectures rely on the integration of multiple sensing, communication, and computing layers. Data are acquired through onboard sensing systems that continuously monitor vehicle operation and surrounding environmental conditions. Modern telemetry platforms collect information from cameras, positioning systems, vehicle dynamics sensors, and communication modules, generating comprehensive datasets that describe both vehicle behavior and external driving conditions. Galinski et al. [4] demonstrated such an approach through a data acquisition platform combining an automotive-grade camera, a high-precision GNSS receiver, and LTE/5G communication modules for real-world driving data collection. The collected information is subsequently transmitted through onboard communication infrastructures that connect sensing devices with local processing units and external communication networks. Similar telemetry solutions have been implemented for Formula SAE vehicles and electric vehicles, enabling continuous performance monitoring, remote diagnostics, and real-time battery management through wireless and IoT-based communication systems [20,22].

Modern telemetry architectures increasingly incorporate real-time processing capabilities. Rather than functioning solely as data collection systems, contemporary platforms provide immediate analysis of operational parameters and support decision-making processes. Farroni et al. [23] introduced a real-time onboard telemetry platform for tire performance evaluation, demonstrating how continuously acquired telemetry information can be used to assess vehicle behavior and operational conditions during driving. Such approaches highlight the transition from passive monitoring systems toward intelligent telemetry environments capable of supporting predictive and adaptive vehicle functions.

**Figure 2:**
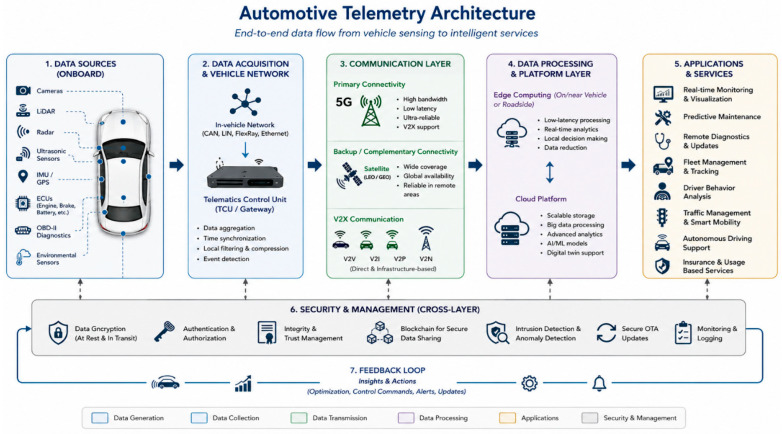
Automotive telemetry architecture and end-to-end data flow in connected vehicles.

The growing volume of telemetry data has also stimulated the development of advanced analytical techniques. Vehicle-generated information is no longer used exclusively for diagnostics but increasingly serves as an input for machine learning and classification algorithms. De Lima et al. [5] demonstrated the application of telemetry-derived data for driver profiling and behavioral classification, illustrating how telemetry systems can provide valuable insights into driving patterns and vehicle usage characteristics. These developments indicate that modern telemetry architectures extend beyond data acquisition and communication, forming the foundation for intelligent transportation analytics. Figure 2 illustrates the end-to-end automotive telemetry architecture used in connected vehicle ecosystems. Data generated by onboard sensors and vehicle systems are collected through telematics units and transmitted via communication networks to edge and cloud platforms for processing and analysis. The resulting information supports a wide range of intelligent mobility services, including predictive maintenance, remote diagnostics, traffic management, and autonomous driving.

Recent developments in connected mobility have further expanded the role of automotive telemetry. The integration of 5G communication technologies enables low-latency and high-reliability transmission of large-scale telemetry datasets between vehicles, infrastructure, and cloud services. Nasreddine et al. [6] demonstrated the implementation of advanced automotive use cases based on real-world 5G networks, including cooperative perception, automotive digital twins, and predictive quality-of-service mechanisms. Such developments illustrate the evolution of automotive telemetry from isolated monitoring systems toward highly interconnected data ecosystems supporting connected and autonomous mobility.

Despite these advances, modern telemetry architectures continue to face challenges related to large-scale data management, interoperability among heterogeneous vehicle platforms, communication reliability, and cybersecurity protection. Addressing these issues will be essential for supporting future intelligent transportation systems and large-scale connected mobility deployments.

### 2.2. Connected Vehicles and Internet of Vehicles

The rapid advancement of communication technologies, cloud computing, and intelligent transportation systems has led to the emergence of connected vehicles as one of the key pillars of modern mobility. Connected vehicles are capable of continuously exchanging information with external entities, including other vehicles, transportation infrastructure, cloud platforms, and mobile devices. This capability extends vehicle awareness beyond the limitations of onboard sensing systems and enables the development of cooperative transportation ecosystems [1,2,3]. According to Abdelkader et al. [1], connected vehicle technologies represent a fundamental shift from isolated transportation systems toward highly interconnected environments where information can be shared and utilized in real time. Ahmed et al. [2] further emphasize that connected and autonomous vehicles are expected to significantly influence future mobility through improvements in transportation safety, efficiency, and sustainability.

Connected vehicles integrate multiple technologies, including wireless communication networks, onboard sensing systems, cloud services, artificial intelligence, and advanced computing platforms. Their architecture typically consists of interconnected layers responsible for data acquisition, communication, processing, and service delivery. Modern vehicles continuously collect operational, environmental, and behavioral data through onboard sensors and electronic control units, which are subsequently transmitted through wireless communication infrastructures for external analysis and utilization in mobility services [3]. Pipicelli et al. [3] describe connected and autonomous vehicles as cyber-physical systems capable of integrating information from both onboard and external sources to support intelligent decision-making processes.

A key element of connected vehicle ecosystems is the Internet of Vehicles (IoV), which extends the Internet of Things paradigm to transportation environments. Within the IoV framework, vehicles function as intelligent network nodes capable of generating, processing, and exchanging large volumes of information. Through continuous connectivity, vehicles communicate with cloud platforms, transportation management systems, roadside infrastructure, and other vehicles, creating distributed networks that support cooperative mobility applications [1,24]. The integration of connected vehicles with IoT technologies further enables advanced services extending beyond transportation itself. Kim et al. [24] demonstrated the feasibility of Home IoT-connected vehicles, where vehicles interact directly with smart home environments, illustrating the growing convergence between mobility services and broader digital ecosystems.

As connected vehicle ecosystems continue to expand, cybersecurity, access control, and data integrity have become increasingly important. The growing number of communication interfaces and connected devices introduces new security vulnerabilities that may compromise system reliability and user privacy. Guan et al. [25] highlighted the importance of cybersecurity frameworks for intelligent connected vehicles, emphasizing secure communication, threat detection, and risk mitigation strategies. Similarly, Ashutosh et al. [26] proposed an access-control framework specifically designed for connected vehicle environments to improve the management of data access and communication permissions. Emerging technologies such as blockchain are also being investigated as mechanisms for improving trust and security within connected mobility ecosystems. Rathee et al. [27] demonstrated how decentralized blockchain-based architectures can enhance authentication, data integrity, and communication security in connected and autonomous vehicles.

Connected vehicle technologies also play a crucial role in supporting cooperative and autonomous driving applications. Kavas-Torris et al. [28] demonstrated the integration of V2X communication between connected and automated vehicles and unmanned aerial vehicles, illustrating how multiple transportation entities can cooperate through real-time information exchange. Similarly, Wang and Peeta [29] investigated cooperative traffic control strategies for connected and autonomous vehicles, highlighting the benefits of coordinated decision-making in complex traffic environments. Additional studies have explored optimization techniques for connected mobility, including communication topology design [30], trajectory prediction mechanisms [31], and energy-efficient operation strategies [32], all of which contribute to improving the performance and sustainability of future transportation systems.

In summary, connected vehicles and the Internet of Vehicles represent a significant evolution in transportation system design. By enabling continuous information exchange among vehicles, infrastructure, cloud platforms, and users, these technologies establish the foundation for intelligent mobility services and future autonomous transportation systems. However, large-scale deployment of IoV ecosystems still faces challenges related to communication reliability, interoperability, cybersecurity protection, data privacy, and management of rapidly growing data volumes. Addressing these challenges will be essential for supporting the next generation of connected, cooperative, and autonomous mobility systems.

### 2.3. Sources and Types of Vehicle Data

The effectiveness of automotive telemetry and connected vehicle systems depends largely on the quantity, quality, and diversity of data generated during vehicle operation. Modern vehicles are equipped with numerous sensors, electronic control units, communication modules, and monitoring systems that continuously collect information describing vehicle performance, driver behavior, environmental conditions, and traffic situations. These data form the foundation of intelligent transportation systems, predictive maintenance solutions, connected mobility services, and autonomous driving technologies [1,2,3].

One of the primary sources of vehicle data originates from onboard sensing systems responsible for monitoring vehicle dynamics and operational parameters. These systems continuously measure variables such as vehicle speed, acceleration, steering angle, braking force, tire pressure, engine operating conditions, and battery status. Galinski et al. [4] demonstrated the collection of real-world driving data using integrated camera systems, GNSS receivers, and telemetry devices, highlighting the increasing capability of modern vehicles to generate comprehensive datasets under various environmental and traffic conditions. Similarly, Farroni et al. [23] utilized real-time telemetry to evaluate tire performance and vehicle behavior, illustrating the importance of continuously monitored operational parameters in automotive applications.

Positioning and navigation systems represent another major category of automotive data sources. Modern vehicles increasingly utilize Global Navigation Satellite Systems (GNSS), including GPS and other positioning technologies, to determine vehicle location, velocity, and trajectory. Such information is essential for navigation services, fleet management, route optimization, and intelligent transportation systems. The dataset presented by Galinski et al. [4] combines positioning information with camera recordings and vehicle telemetry, demonstrating how multiple data sources can be integrated to provide a comprehensive representation of vehicle operation within real traffic environments.

The growing adoption of advanced driver assistance systems (ADAS) has significantly expanded the volume and complexity of vehicle-generated information. Cameras, radar sensors, LiDAR units, ultrasonic sensors, and inertial measurement systems continuously monitor the surrounding environment and provide information regarding nearby vehicles, pedestrians, traffic infrastructure, and road conditions. These data support functions such as lane-keeping assistance, adaptive cruise control, collision avoidance, and autonomous driving. As connected vehicles evolve, environmental perception data have become among the most data-intensive components of modern automotive telemetry architectures [1,2,3].

**Figure 3:**
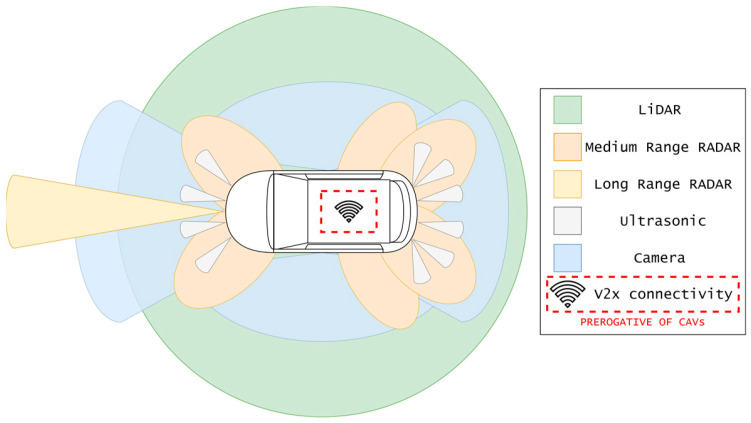
Typical sensor configuration and data acquisition sources in connected vehicles [3].

Figure 3 illustrates a typical sensor configuration used in connected and autonomous vehicles. Multiple sensing technologies operate simultaneously to provide comprehensive environmental perception. LiDAR sensors offer high-resolution three-dimensional mapping of the vehicle surroundings, radar systems support object detection and distance estimation under varying weather conditions, cameras provide visual information for object recognition and lane detection, while ultrasonic sensors are primarily used for short-range obstacle detection. In addition to onboard sensing systems, V2X communication extends situational awareness beyond the physical sensing range by enabling the exchange of information with other vehicles, roadside infrastructure, and transportation networks. The integration of these complementary technologies forms the basis of modern automotive telemetry and supports advanced driver assistance and autonomous driving functions.

Vehicle-generated information can generally be categorized into operational, diagnostic, behavioral, safety-related, and simulation-derived datasets. Operational and diagnostic data include vehicle performance indicators, energy consumption, battery condition, fault codes, and system health information, supporting monitoring, optimization, and predictive maintenance activities [22,30]. Driver behavior data, such as acceleration patterns, braking behavior, steering inputs, and route preferences, can be utilized for driver profiling, fleet management, and personalized mobility services. De Lima et al. [5] demonstrated the application of classification algorithms to telemetry-derived driver data, enabling the identification of distinct driver profiles and behavioral patterns. Safety-related datasets include accident records, emergency braking events, collision warnings, and airbag deployment information. He et al. [33] demonstrated the value of crash telemetry data for predicting injury severity following vehicle collisions, highlighting the importance of telemetry systems in emergency response and post-crash assessment. In addition, simulation and virtual testing environments provide valuable synthetic datasets for vehicle development, validation, and digital twin applications. Dias et al. [34] demonstrated the use of dynamic vehicle simulation for braking system analysis, illustrating the growing role of simulation-generated data in modern automotive engineering.

As connected vehicle ecosystems continue to evolve, data generated by individual vehicles are increasingly integrated with information obtained from infrastructure systems, cloud platforms, and communication networks. This integration enables the creation of comprehensive mobility datasets capable of supporting intelligent transportation services, traffic management systems, autonomous driving functions, and predictive analytics. Consequently, modern automotive telemetry has evolved from a simple monitoring tool into a complex data ecosystem where information from multiple sources is continuously collected, transmitted, analyzed, and utilized to improve transportation safety, efficiency, and sustainability [1,2,3].

Despite the substantial benefits provided by large-scale vehicle-generated datasets, several challenges remain. Data heterogeneity, sensor reliability, privacy protection, synchronization of multiple data sources, and efficient processing of high-volume perception data continue to represent major obstacles for large-scale deployment of connected mobility services. These challenges are expected to become increasingly significant with the growing adoption of autonomous driving technologies, artificial intelligence, and digital twin applications.

The increasing diversity and volume of vehicle-generated data place significant demands on communication infrastructures responsible for transmitting information between vehicles, cloud services, and transportation systems. Therefore, reliable and high-capacity communication technologies have become essential components of connected mobility ecosystems and are discussed in the following chapter.

When the reviewed telemetry architectures are considered comparatively, several trade-offs emerge that are rarely made explicit in the primary literature. A first tension concerns the placement of data processing: transmitting raw sensor streams to the cloud maximizes analytical flexibility but is economically and technically unrealistic at fleet scale, whereas aggressive onboard preprocessing reduces communication load at the cost of discarding information that may later prove valuable for diagnostics or model training; the reviewed studies adopt widely different operating points on this spectrum without justifying them quantitatively. Second, there is still no widely adopted standard for telemetry data models and access interfaces—initiatives such as the ISO 20077/20078 extended-vehicle framework coexist with numerous proprietary manufacturer platforms—which fragments the ecosystem and complicates cross-brand fleet analytics [26]. Third, telemetry datasets are inherently privacy-sensitive, since location and driving-behavior traces can allow the re-identification of individual drivers, yet only a minority of the reviewed works address anonymization, consent management, or regulatory compliance [5,26]. These unresolved aspects should be regarded as prerequisites for, rather than accessories to, the large-scale telemetry deployments discussed in the following sections.

## 3. Communication Technologies for Long-Range Data Transmission

The continuous growth of connected vehicle ecosystems has significantly increased the demand for reliable, high-capacity, and low-latency communication infrastructures. Modern vehicles continuously generate large volumes of telemetry data that must be transmitted between onboard systems, cloud platforms, transportation management centers, infrastructure elements, and other vehicles. Consequently, the effectiveness of automotive telemetry depends not only on the quality of data acquisition but also on the ability of communication networks to deliver information efficiently, securely, and in real time.

Traditional wireless communication technologies were primarily designed to support human-to-human communication and often struggle to satisfy the requirements of modern intelligent transportation systems. Automotive applications frequently require ultra-low latency, high reliability, massive device connectivity, and uninterrupted operation across geographically diverse environments. These requirements are particularly critical for autonomous driving, cooperative mobility, predictive maintenance, fleet management, and safety-critical decision-making processes.

As transportation systems become increasingly data-driven, communication infrastructures have evolved into one of the key enabling technologies of connected mobility. Among the available communication solutions, fifth-generation mobile communication networks (5G) and satellite communication systems have emerged as the most promising technologies for long-range automotive data transmission. While 5G networks provide high bandwidth, low latency, and support for large-scale connectivity in urban and suburban environments, satellite communication systems offer extensive geographical coverage and enable communication in regions where terrestrial infrastructures may be unavailable or insufficient.

Despite substantial progress in communication technologies, no single communication platform currently satisfies all requirements of connected mobility. Consequently, research increasingly focuses on hybrid communication architectures that combine terrestrial and non-terrestrial communication infrastructures to provide seamless connectivity across diverse transportation scenarios while mitigating the limitations of individual technologies.

This chapter examines the fundamental principles, architectures, advantages, limitations, and practical applications of modern communication technologies used in automotive telemetry. Attention is particularly devoted to 5G communication systems, satellite communication technologies, and emerging hybrid architectures that support future connected mobility services.

### 3.1. 5G Communication Technologies

The increasing volume of data generated by connected vehicles has created new demands on communication infrastructures capable of supporting real-time information exchange. Traditional cellular technologies often struggle to meet the stringent requirements associated with intelligent transportation systems, autonomous driving, and large-scale telemetry applications. Consequently, fifth-generation (5G) communication networks have emerged as a key enabling technology for future connected mobility ecosystems. Their ability to provide high data rates, low latency, massive device connectivity, and enhanced reliability makes them particularly suitable for automotive applications [7,35].

The role of 5G within intelligent transportation systems extends far beyond conventional mobile communication services. Gohar and Nencioni [7] emphasize that 5G technologies provide the communication foundation necessary for supporting smart city infrastructures and next-generation transportation systems. Compared with previous generations of cellular networks, 5G enables substantially faster information exchange, improved network responsiveness, and the simultaneous connection of large numbers of devices. These capabilities are particularly important in transportation environments where vehicles, infrastructure components, pedestrians, and cloud platforms continuously exchange operational and safety-related information.

A major advantage of 5G technology is its support for Vehicle-to-Everything (V2X) communication. Modern transportation systems increasingly rely on cooperative information exchange among vehicles and external entities to improve situational awareness and traffic efficiency. Condoluci et al. [8] proposed a system-level architecture for 5G-enabled V2X communication, demonstrating how next-generation cellular networks can support cooperative mobility services. Similarly, Chatzoulis et al. [36] investigated the performance of 3GPP-compliant 5G V2X communication under various propagation environments, highlighting the importance of reliable communication mechanisms for maintaining quality-of-service requirements in connected transportation systems.

**Figure 4:**
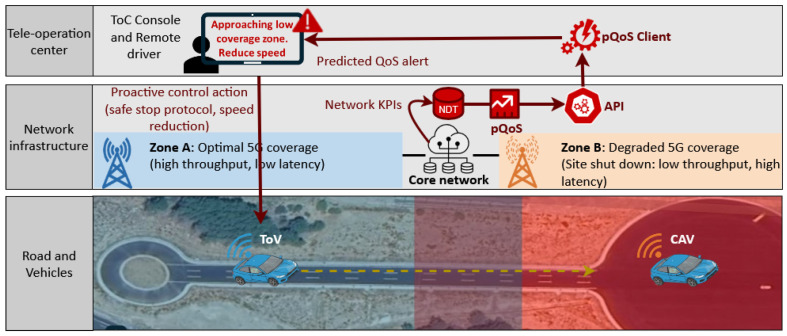
Predictive quality-of-service management in 5G-enabled connected vehicles [6]. The blue and orange shaded areas indicate Zone A (optimal 5G coverage) and Zone B (degraded 5G coverage), respectively, while the red overlay on the road marks the segment affected by the degraded coverage.

Figure 4 illustrates a predictive Quality of Service (QoS) management framework for 5G-enabled connected and autonomous vehicles. The architecture continuously monitors network key performance indicators (KPIs), such as latency, throughput, and coverage quality, to anticipate potential communication degradations before they affect vehicle operation. When a vehicle approaches an area with reduced network coverage, the system generates a predictive QoS alert and initiates proactive control actions, including speed reduction or safe-stop procedures. By combining real-time network analytics, edge intelligence, and V2X communication, such approaches enhance communication reliability and support the safe operation of connected and autonomous vehicles in dynamic transportation environments. This capability is particularly important for safety-critical applications where uninterrupted connectivity and low latency are essential for decision-making processes.

The deployment of Multi-access Edge Computing (MEC) has further enhanced the capabilities of 5G communication networks. Traditional cloud-based architectures often introduce communication delays that may be unacceptable for safety-critical automotive applications. MEC addresses this challenge by relocating computational resources closer to end users and data sources. Wadatkar et al. [37] demonstrated the use of 5G-MEC testbeds for V2X applications, showing how edge computing can reduce latency and improve communication efficiency. The integration of edge intelligence with 5G infrastructures enables real-time data processing and supports emerging mobility services that require rapid decision-making and localized analytics.

Reliable wireless communication in automotive environments depends heavily on advanced antenna and beamforming technologies. Modern connected vehicles require compact, efficient, and wideband antenna systems capable of maintaining stable communication under dynamic operating conditions. Michel et al. [38] developed a compact dashboard antenna optimized for vehicular LTE and 5G communication, while Tayyab et al. [39] proposed a dual-band circularly polarized antenna array for automotive satellite and 5G applications. In addition, beamforming technologies improve signal quality, spectrum utilization, and communication efficiency by directing wireless signals toward specific users. Hussain and Oh [40] demonstrated how integrated radar and communication systems can simultaneously support sensing and communication functionalities, highlighting the growing convergence of perception and connectivity within intelligent transportation systems.

As transportation systems become increasingly dependent on wireless connectivity, cybersecurity has emerged as an important challenge for 5G-enabled mobility services. The growing use of connected devices, cloud services, and over-the-air software updates increases the potential attack surface available to malicious actors. Oh et al. [41] investigated security threats associated with 5G firmware over-the-air update mechanisms, while Algarni and Thayananthan [42] proposed software-defined networking-based security solutions for protecting transportation services. Additional research by Niranjan et al. [43] demonstrated secure data transmission approaches within Internet of Vehicles environments using 5G communication infrastructures.

Recent developments indicate that the future of connected mobility will increasingly depend on the convergence of 5G communication, artificial intelligence, edge computing, and autonomous vehicle technologies. Biswas and Wang [9] described a framework integrating IoT, edge intelligence, blockchain, and 5G communication to support autonomous vehicle ecosystems. Such integrated architectures enable intelligent data processing, distributed decision-making, and secure information exchange while supporting the growing complexity of connected transportation systems.

Despite its significant advantages, 5G technology still faces several limitations in automotive environments. Network coverage may be constrained in rural and remote regions, while mmWave frequencies remain highly susceptible to signal attenuation caused by obstacles, weather conditions, and vehicle mobility. Furthermore, large-scale deployment of 5G infrastructures requires substantial investments and complex coordination among communication providers, transportation authorities, and vehicle manufacturers. These challenges have motivated increasing interest in complementary communication technologies, particularly satellite and hybrid terrestrial–non-terrestrial architectures.

Taken together, 5G communication technologies represent a fundamental component of future intelligent mobility ecosystems. Their ability to support low-latency communication, high data throughput, edge-based processing, and large-scale device connectivity provides the foundation for advanced automotive telemetry, V2X communication, autonomous driving, and smart transportation services. As deployment continues to expand globally, 5G is expected to become one of the key technologies enabling the transition toward fully connected and autonomous mobility environments.

### 3.2. Satellite Communication Technologies

Despite the rapid deployment of terrestrial communication infrastructures, maintaining reliable connectivity across all transportation environments remains a significant challenge. Many rural areas, remote transportation corridors, maritime routes, and disaster-affected regions lack sufficient terrestrial network coverage to support continuous data transmission. Satellite communication technologies address this limitation by providing large-scale geographical coverage and enabling communication services in locations where conventional cellular infrastructures are unavailable or impractical. Consequently, satellite systems have become increasingly important components of modern connected mobility ecosystems [10,11,12].

Satellite communication systems provide several advantages for intelligent transportation applications. Their primary benefit lies in the ability to deliver communication services across extensive geographical regions regardless of local infrastructure availability. This capability is particularly valuable for long-distance transportation, logistics operations, emergency response services, and autonomous mobility applications operating beyond the coverage of terrestrial communication networks. As connected vehicles continue to generate increasing volumes of telemetry data, satellite systems offer an effective solution for ensuring communication continuity across diverse operating environments [11,12].

The growing importance of satellite communication has stimulated significant technological development within the field. Modern satellite networks are evolving toward more flexible, intelligent, and adaptive architectures capable of supporting diverse communication requirements. Zeng et al. [44] proposed an artificial intelligence-based approach for predicting service demands in satellite communication terminals, while Lee [45] demonstrated the application of reinforcement learning techniques for frequency hopping synchronization within satellite communication networks. These studies illustrate the growing role of machine learning in resource allocation, synchronization, communication robustness, and network optimization.

Optical communication technologies have also emerged as promising alternatives to conventional radio-frequency satellite links. Free Space Optical (FSO) communication enables high-capacity data transmission using optical signals rather than radio waves. Marbel et al. [46] investigated dynamic network formation strategies for FSO satellite communication systems, demonstrating the potential of optical communication technologies to support high-bandwidth satellite networks. Similarly, Palmer and Kulau [47] developed and evaluated a LiFi-based transceiver module for telemetry, tracking, and command communication within satellite environments. Together, these developments highlight the growing importance of optical wireless communication technologies in future satellite architectures.

Satellite communication technologies also play an important role in disaster response and emergency management applications. During natural disasters or large-scale infrastructure failures, terrestrial communication networks may become unavailable or severely degraded. Sui et al. [48] investigated the scheduling of satellite communication vehicles for post-disaster operations, highlighting the importance of satellite-based communication systems for maintaining connectivity and supporting critical infrastructure recovery efforts.

The emergence of small satellites and CubeSat platforms has further accelerated innovation within the satellite communication sector. CubeSat technologies offer cost-effective alternatives to traditional satellite systems while enabling rapid deployment of communication services. Akyildiz et al. [49] proposed a CubeSat architecture incorporating reconfigurable multi-band radios capable of supporting dynamic spectrum management, illustrating the growing flexibility of next-generation satellite communication systems.

Recent developments in Non-Terrestrial Networks (NTNs) have created new opportunities for integrating satellite communication into future mobile communication ecosystems. NTNs extend conventional terrestrial communication infrastructures by incorporating satellites and other non-terrestrial platforms into unified communication architectures. Borshch et al. [50] investigated timing synchronization challenges associated with Orthogonal Time Frequency Space (OTFS) communication in NTN scenarios, highlighting the technical requirements necessary for supporting future satellite-integrated communication systems.

The convergence of satellite communication and 5G technologies represents one of the most significant developments in contemporary communication research. Völk et al. [11] demonstrated the integration of satellite backhaul capabilities into 5G communication architectures, while Uko et al. [12] investigated performance optimization strategies for integrated 5G-satellite networks supporting Internet of Things applications. These studies illustrate the growing trend toward hybrid communication infrastructures capable of combining the strengths of terrestrial and satellite technologies to provide reliable, large-scale connectivity.

Despite their extensive coverage and strategic importance, satellite communication systems also face several limitations. Compared with terrestrial 5G networks, satellite links often experience higher latency, limited bandwidth availability, and increased sensitivity to atmospheric conditions. Furthermore, the deployment and operation of satellite infrastructures involve substantial economic and technical challenges, particularly in highly dynamic communication environments. These limitations have motivated ongoing research into hybrid terrestrial–non-terrestrial communication architectures that seek to combine the global coverage of satellites with the low-latency performance of terrestrial networks [12].

Collectively, satellite communication technologies continue to evolve from standalone communication systems toward intelligent, adaptive, and highly integrated network architectures. Advances in artificial intelligence, optical communication, CubeSat platforms, and Non-Terrestrial Networks are expanding the capabilities of satellite communication while improving performance, flexibility, and scalability. As future transportation systems become increasingly dependent on continuous connectivity, satellite communication is expected to play an essential role in supporting global mobility services, intelligent transportation systems, and next-generation connected vehicle ecosystems [50].

The integration of satellite systems into mainstream mobile networks has been formalized through the 3GPP standardization of Non-Terrestrial Networks (NTN). Release 17 (2022) introduced the first normative NTN specifications, defining NR-NTN for broadband services and IoT-NTN for narrowband applications, both based on transparent (“bent-pipe”) payload architectures in which the satellite acts as a radio repeater while the gNB remains on the ground (3GPP TR 38.821 [51]). Release 18 (5G-Advanced) extended these specifications with coverage and mobility enhancements, including support for discontinuous coverage relevant to sparse LEO constellations, while the ongoing Release 19 work introduces regenerative payloads that place a complete gNB on board the satellite, enabling tighter integration between terrestrial and non-terrestrial segments. For automotive telemetry, this standardization trajectory is significant because it allows satellite connectivity to be provided through standardized, mass-market 5G chipsets rather than proprietary satellite terminals, substantially reducing terminal cost and integration complexity [11].

The choice of orbital regime has direct consequences for telemetry applications. Geostationary (GEO) satellites at approximately 35,786 km provide continuous regional coverage from a single satellite and require no handover management; however, round-trip propagation delays of roughly 480–560 ms make GEO links unsuitable for latency-critical vehicular services and limit their role to delay-tolerant telemetry, fleet reporting, and broadcast services. Low Earth Orbit (LEO) constellations operating at 500–2000 km reduce round-trip latencies to approximately 30–50 ms, which is sufficient for most telemetry and many near-real-time applications, but they introduce new challenges: individual satellite visibility windows last only a few minutes, requiring frequent handovers; large constellations are needed to guarantee service continuity; and high relative velocities produce significant Doppler shifts that must be compensated at the physical layer. Medium Earth Orbit (MEO) systems represent an intermediate trade-off between these extremes. Consequently, the literature increasingly converges on the view that LEO constellations constitute the most suitable non-terrestrial component for vehicular services, while GEO systems retain value for wide-area, delay-tolerant data collection [12].

Mobility management constitutes one of the most demanding aspects of NTN-based vehicular connectivity, since handovers are driven not only by vehicle movement but primarily by satellite motion. 3GPP Releases 17 and 18 therefore introduced ephemeris-assisted and location-based mobility mechanisms, conditional handover procedures, and support for both Earth-fixed and Earth-moving beam configurations. For connected vehicles, the combination of terminal mobility and constellation dynamics creates a dual-mobility problem that remains insufficiently validated in real automotive scenarios, particularly with respect to service continuity during frequent inter-satellite and satellite-to-terrestrial handovers [11,12].

At the level of individual 3GPP releases, the NTN feature set relevant to automotive telemetry can be summarized as follows. Release 17 defined the fundamental adaptations of the NR air interface to satellite channels, including autonomous timing-advance and frequency pre-compensation based on broadcast satellite ephemeris, extended HARQ operation for long propagation delays, and the IoT-NTN profile for NB-IoT/eMTC traffic, whose small message sizes match periodic telemetry reporting. Release 18 (5G-Advanced) added coverage enhancements for handheld-class terminals, support for NTN operation above 10 GHz (Ka-band) for higher-throughput services, network-verified terminal location, and improved mobility between terrestrial and non-terrestrial cells. Release 19 extends the framework with regenerative payloads, store-and-forward operation for delay-tolerant IoT-NTN traffic—directly applicable to telemetry collection from vehicles outside continuous feeder-link coverage—and support for reduced-capability (RedCap) devices, which lowers the cost and power consumption of vehicle-mounted NTN modems [11,12,50,51].

The practical severity of the dual-mobility problem can be illustrated quantitatively. A LEO satellite at an altitude of 600 km moves at approximately 7.6 km/s relative to the Earth’s surface, so a single satellite remains visible to a vehicle for only about 4–8 min per pass, and service-link handovers must consequently be executed every few minutes even for a stationary terminal; vehicle motion at highway speeds is almost negligible by comparison, but it adds measurement and beam-management complexity. Doppler shifts of up to roughly ±24 ppm of the carrier frequency (about ±48 kHz at 2 GHz) must be pre-compensated using ephemeris data. To keep signaling overhead manageable, 3GPP introduced conditional handover, in which candidate target cells are prepared in advance and the handover is executed autonomously by the terminal once time- or location-based conditions are met, together with Earth-fixed beam operation, in which beams are steered to illuminate a fixed geographical area during a satellite pass. Feeder-link switchover—the migration of an entire satellite from one ground gateway to another—introduces an additional, less frequently studied interruption source. For automotive telemetry, the key open question is therefore not whether individual handovers succeed, but whether session continuity and bounded latency can be maintained across the concatenation of intra-constellation, inter-constellation, and satellite-to-terrestrial handovers experienced during a long-distance journey [11,12,50,51].

To make the orbital trade-offs discussed above explicit, Table 2 summarizes the quantitative characteristics of the principal orbital regimes and of high-altitude platform systems (HAPS) from the perspective of automotive telemetry.

### 3.3. Hybrid 5G–Satellite Architectures

The increasing demand for continuous connectivity in modern transportation systems has revealed the limitations of relying solely on either terrestrial or satellite communication infrastructures. While 5G networks provide ultra-low latency, high bandwidth, and support for real-time applications, their performance remains dependent on the availability of terrestrial communication infrastructure. In contrast, satellite communication systems offer extensive geographical coverage but often experience higher latency and lower data transmission rates compared with terrestrial networks. Consequently, hybrid 5G–satellite architectures have emerged as a promising solution for future connected mobility ecosystems by combining the complementary strengths of both communication technologies [11,12].

**Figure 5:**
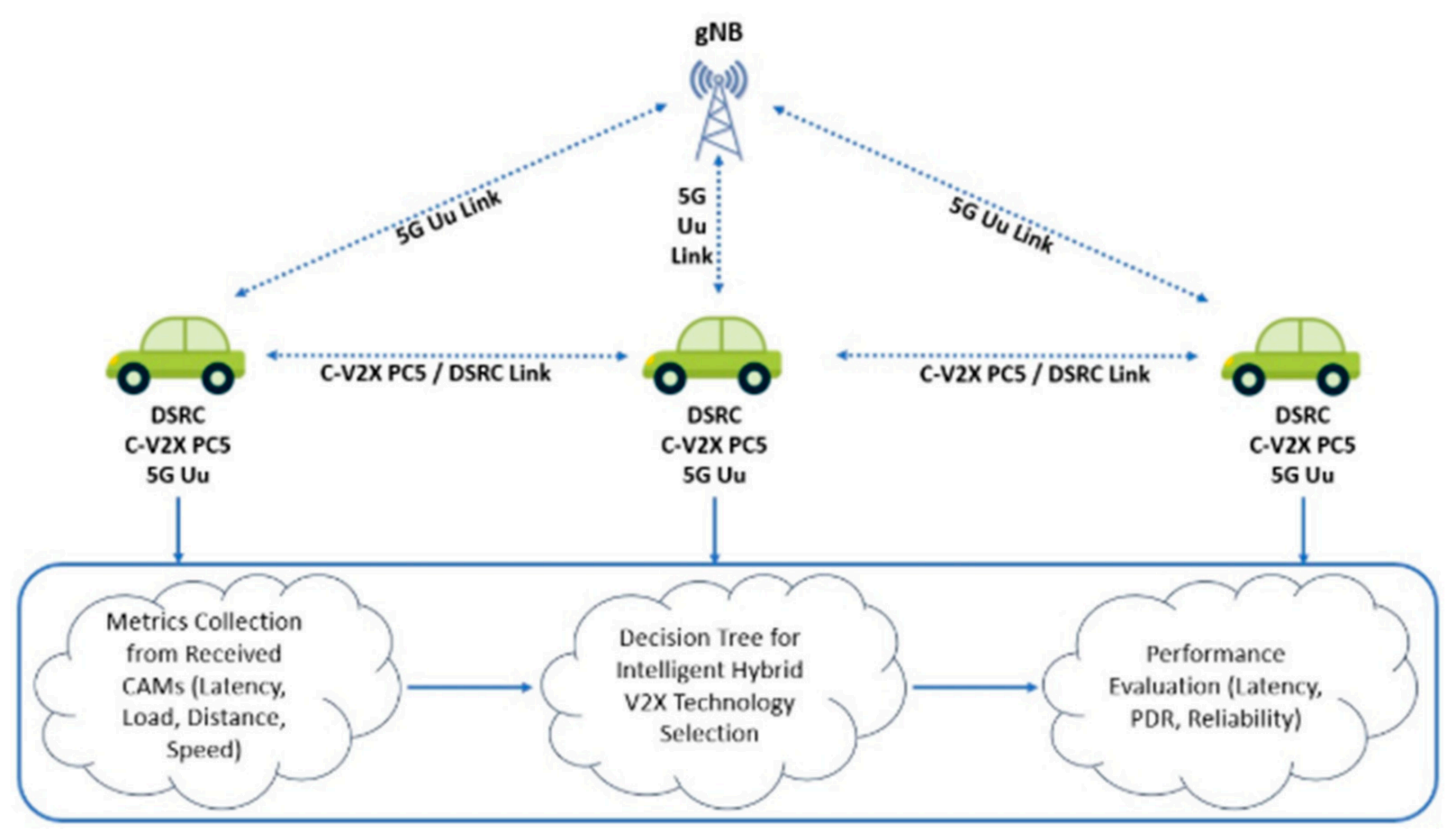
Intelligent hybrid V2X communication technology selection framework based on network performance metrics and communication requirements [8].

The primary objective of hybrid communication architectures is to ensure seamless and uninterrupted connectivity regardless of vehicle location or operating environment. In urban and suburban areas, vehicles can primarily utilize terrestrial 5G networks to support latency-sensitive applications such as autonomous driving, cooperative perception, vehicle-to-everything communication, and real-time telemetry transmission. When vehicles move beyond terrestrial network coverage, communication services can be transferred to satellite networks, maintaining connectivity and enabling continuous access to critical transportation services [11]. Figure 5 presents an intelligent hybrid communication framework designed to dynamically select the most suitable V2X communication technology according to current network conditions and application requirements. The architecture collects communication performance metrics, including latency, vehicle speed, transmission distance, channel load, and packet delivery reliability. Based on these parameters, a decision-making algorithm evaluates the available communication technologies, such as DSRC, C-V2X PC5, and 5G cellular links, and selects the optimal communication path for a given scenario. Such adaptive communication management improves network efficiency, enhances communication reliability, and supports the diverse quality-of-service requirements of connected and autonomous vehicle applications. Intelligent hybrid communication strategies are expected to play a key role in future transportation systems, where heterogeneous communication infrastructures must operate cooperatively to provide robust and uninterrupted connectivity.

The integration of satellite communication into 5G infrastructures has become one of the major research directions within next-generation communication systems. Völk et al. [11] demonstrated the feasibility of integrating satellite backhaul capabilities into 5G architectures through over-the-air testing of edge node concepts, while Uko et al. [12] investigated performance optimization strategies for integrated 5G–satellite networks supporting IoT applications. These studies highlight how hybrid communication infrastructures can improve network resilience, service availability, and coverage while supporting large-scale deployments of connected devices and mobility services.

Hybrid communication systems are particularly important for highly dynamic transportation environments in which vehicles frequently operate across regions with varying communication coverage conditions. Velez et al. [35] emphasized the importance of advanced 5G communication capabilities for connected automated mobility applications operating across national and regional boundaries. Similarly, Biswas and Wang [9] proposed an integrated framework combining IoT technologies, edge intelligence, blockchain, and 5G communication to support autonomous vehicle ecosystems. The incorporation of satellite communication capabilities into such architectures can further enhance system resilience and facilitate large-scale deployment of autonomous transportation services.

The integration of advanced antenna technologies also contributes to the successful implementation of hybrid communication systems. Tayyab et al. [39] developed a dual-band circularly polarized antenna array specifically designed for automotive satellite and 5G communication applications. Such antenna systems enable vehicles to communicate efficiently with both terrestrial and satellite networks while maintaining stable connectivity under dynamic operating conditions. The development of multi-band communication hardware therefore represents an important enabling technology for future hybrid communication architectures.

Despite their considerable potential, hybrid 5G–satellite systems face several technical challenges. Network synchronization, communication handover procedures, resource allocation strategies, latency management, and interoperability among heterogeneous communication infrastructures remain active areas of research. Furthermore, maintaining consistent quality of service across communication technologies with different performance characteristics requires sophisticated network management mechanisms capable of dynamically selecting the most appropriate communication resources for each application. Despite their advantages, hybrid architectures inevitably introduce additional system complexity, requiring advanced orchestration mechanisms capable of managing multiple communication technologies simultaneously.

The emergence of Non-Terrestrial Networks and future 6G communication concepts is expected to further strengthen the integration between terrestrial and satellite communication infrastructures. Rather than functioning as separate communication systems, future networks are likely to operate as unified communication ecosystems where terrestrial cellular networks, satellite constellations, aerial platforms, and edge computing resources cooperate to provide ubiquitous connectivity. Such developments will play a critical role in supporting autonomous mobility, intelligent transportation systems, digital twins, and next-generation connected vehicle services.

Viewed as a whole, hybrid 5G–satellite architectures represent one of the most promising approaches for achieving reliable, large-scale, and continuous connectivity within future transportation ecosystems. By combining the low latency and high throughput of terrestrial 5G networks with the extensive coverage offered by satellite communication systems, these architectures establish a robust communication foundation capable of supporting the growing demands of connected and autonomous mobility.

Beyond radio-level integration, hybrid architectures raise system-level questions of resource management and service differentiation. Network slicing enables the logical separation of vehicular services with conflicting requirements—for example, an ultra-reliable low-latency slice for safety-related V2X messaging, an enhanced mobile broadband slice for infotainment, and a massive machine-type communication slice for periodic telemetry reporting. Extending slice management consistently across terrestrial and non-terrestrial segments, however, remains an open research problem, since satellite resources are scarcer and subject to different dynamics than terrestrial spectrum. Closely related are QoS and QoE management mechanisms: predictive quality-of-service approaches, such as those demonstrated by Nasreddine et al. [6], allow applications to adapt proactively to anticipated connectivity degradations, which is particularly valuable at the boundary between terrestrial coverage and satellite fallback.

In the 5G system, this service differentiation is formalized through standardized 5G QoS identifiers (5QI): the dedicated V2X service classes specified in 3GPP TS 23.287 [52] define packet delay budgets between 10 and 100 ms with reliability targets up to 99.999%, against which any non-terrestrial segment must be benchmarked. The comparison in Table 2 shows that only LEO systems approach the delay budgets of the less stringent V2X service classes, while GEO systems fall outside all of them, confirming that satellite segments should primarily carry delay-tolerant telemetry and leave time-critical safety messaging to terrestrial links. Beyond network-level QoS, quality of experience (QoE) becomes the relevant metric for infotainment and teleoperation services, where session continuity during vertical handovers influences perceived quality more strongly than average throughput; systematic QoE studies for hybrid terrestrial/non-terrestrial vehicular links are, however, still largely missing from the literature [8,11,36].

A further architectural dimension concerns the placement of computation. Multi-access edge computing (MEC) brings processing capacity close to the radio access network, reducing backhaul load and end-to-end latency for telemetry analytics, cooperative perception, and digital twin synchronization. In vehicular environments, computation offloading decisions must additionally account for mobility, fluctuating channel quality, and intermittent coverage; recent research on vehicular edge computing therefore addresses the joint optimization of communication and computation resources, including emerging concepts such as fluid antenna-assisted MEC networks that jointly consider channel estimation and computation offloading [6]. In hybrid terrestrial/non-terrestrial systems, the advent of regenerative satellite payloads additionally opens the possibility of satellite edge computing, in which selected processing tasks are executed on board the satellite, although the energy and computational constraints of space platforms currently limit this approach to lightweight workloads [6].

When the reviewed communication technologies are compared critically, several tensions become apparent. First, the long-standing competition between IEEE 802.11p/DSRC and cellular C-V2X has produced conflicting experimental findings—studies emphasizing mature, low-cost deployments tend to favor DSRC, whereas studies emphasizing evolution paths toward 5G NR sidelink favor C-V2X—and regulatory decisions have diverged across regions, with China committing to C-V2X, the United States reallocating most of the 5.9 GHz band, and Europe long maintaining technology neutrality. Second, there is no consensus in the literature on whether LEO constellations can support safety-critical V2X services, or whether their role should remain limited to non-safety telemetry: reported end-to-end latencies vary widely depending on constellation design, gateway placement, and traffic assumptions. Third, quantitative cost analyses of hybrid architectures are almost entirely absent from the reviewed literature, which makes techno-economic feasibility one of the least understood aspects of the field. These unresolved trade-offs should be considered when interpreting the comparison presented in Table 3.

The communication infrastructure of connected vehicles is based on several complementary technologies, each offering distinct advantages and limitations in terms of coverage, latency, throughput, and deployment requirements. A comparison of the most commonly used communication technologies in connected mobility ecosystems is presented in Table 3.

As shown in Table 3, no single communication technology can fully satisfy all connected mobility requirements. Consequently, future transportation systems are expected to increasingly rely on hybrid communication architectures that combine the strengths of terrestrial and non-terrestrial communication technologies.

## 4. Vehicle-to-Everything (V2X) Communication and Applications

The increasing complexity of connected transportation systems has accelerated the development of communication paradigms capable of supporting direct information exchange among vehicles, infrastructure components, pedestrians, cloud services, and other transportation entities. One of the most important concepts emerging from this evolution is Vehicle-to-Everything (V2X) communication. V2X represents a comprehensive communication framework that enables vehicles to exchange information with their surrounding environment in real time, thereby improving safety, traffic efficiency, situational awareness, and overall transportation system performance.

Traditional vehicle operation primarily relies on onboard sensors such as cameras, radar systems, LiDAR units, and ultrasonic sensors to perceive the surrounding environment. Although these technologies provide valuable information regarding nearby objects and traffic conditions, their effectiveness is inherently limited by sensor range, line-of-sight constraints, weather conditions, and environmental obstacles. V2X communication extends vehicle perception by enabling access to information generated by external entities, allowing vehicles to obtain awareness of events occurring beyond the reach of onboard sensing systems and make more informed operational decisions.

The fundamental objective of V2X communication is to facilitate continuous information exchange among transportation system participants. Through real-time communication, vehicles can share information regarding their position, velocity, acceleration, intended trajectory, vehicle status, road conditions, and detected hazards. This cooperative information-sharing approach enables transportation systems to utilize both local observations and externally acquired knowledge, thereby improving decision-making processes and transportation efficiency.

V2X communication encompasses several communication categories, including Vehicle-to-Vehicle (V2V), Vehicle-to-Infrastructure (V2I), Vehicle-to-Network (V2N), Vehicle-to-Pedestrian (V2P), and Vehicle-to-Cloud (V2C) communication. Together, these communication modes form an integrated framework capable of supporting a wide range of intelligent transportation applications. Their effectiveness depends heavily on the availability of reliable communication infrastructures capable of supporting low-latency and high-reliability data exchange. Modern V2X systems increasingly utilize 5G communication technologies, edge computing platforms, cloud services, and hybrid communication architectures to ensure rapid information dissemination and continuous connectivity.

Beyond improving road safety, V2X communication contributes significantly to traffic efficiency, environmental sustainability, and autonomous mobility. By enabling vehicles to cooperate and exchange information regarding traffic conditions, route availability, and infrastructure status, transportation systems can reduce congestion, improve traffic flow, decrease travel times, and optimize energy consumption. Furthermore, V2X provides autonomous vehicles with additional layers of information that complement onboard sensing technologies and enhance perception accuracy, situational awareness, and decision-making capabilities.

The continuous evolution of V2X communication technologies has stimulated the development of new applications involving artificial intelligence, blockchain technologies, edge computing, and digital transportation infrastructures. These innovations contribute to the creation of increasingly intelligent and adaptive mobility ecosystems capable of responding dynamically to changing transportation conditions. Despite its considerable potential, the effectiveness of V2X communication remains dependent on communication reliability, interoperability among heterogeneous systems, cybersecurity protection, and large-scale infrastructure deployment.

Given its central role in future transportation systems, a detailed examination of the various V2X communication categories and their practical applications is essential. Therefore, the following sections discuss the major V2X communication modes, their operational principles, technological requirements, and contributions to intelligent mobility and connected vehicle ecosystems.

### 4.1. Types of V2X Communication

Vehicle-to-Everything (V2X) communication represents one of the fundamental enabling technologies for connected and autonomous mobility. The concept refers to a communication framework that allows vehicles to exchange information with various entities within the transportation ecosystem, including other vehicles, roadside infrastructure, pedestrians, cloud services, and communication networks. By extending situational awareness beyond the limitations of onboard sensors, V2X communication enhances transportation safety, traffic efficiency, and operational reliability while supporting the development of intelligent transportation systems and autonomous driving applications [13,14].

**Figure 6:**
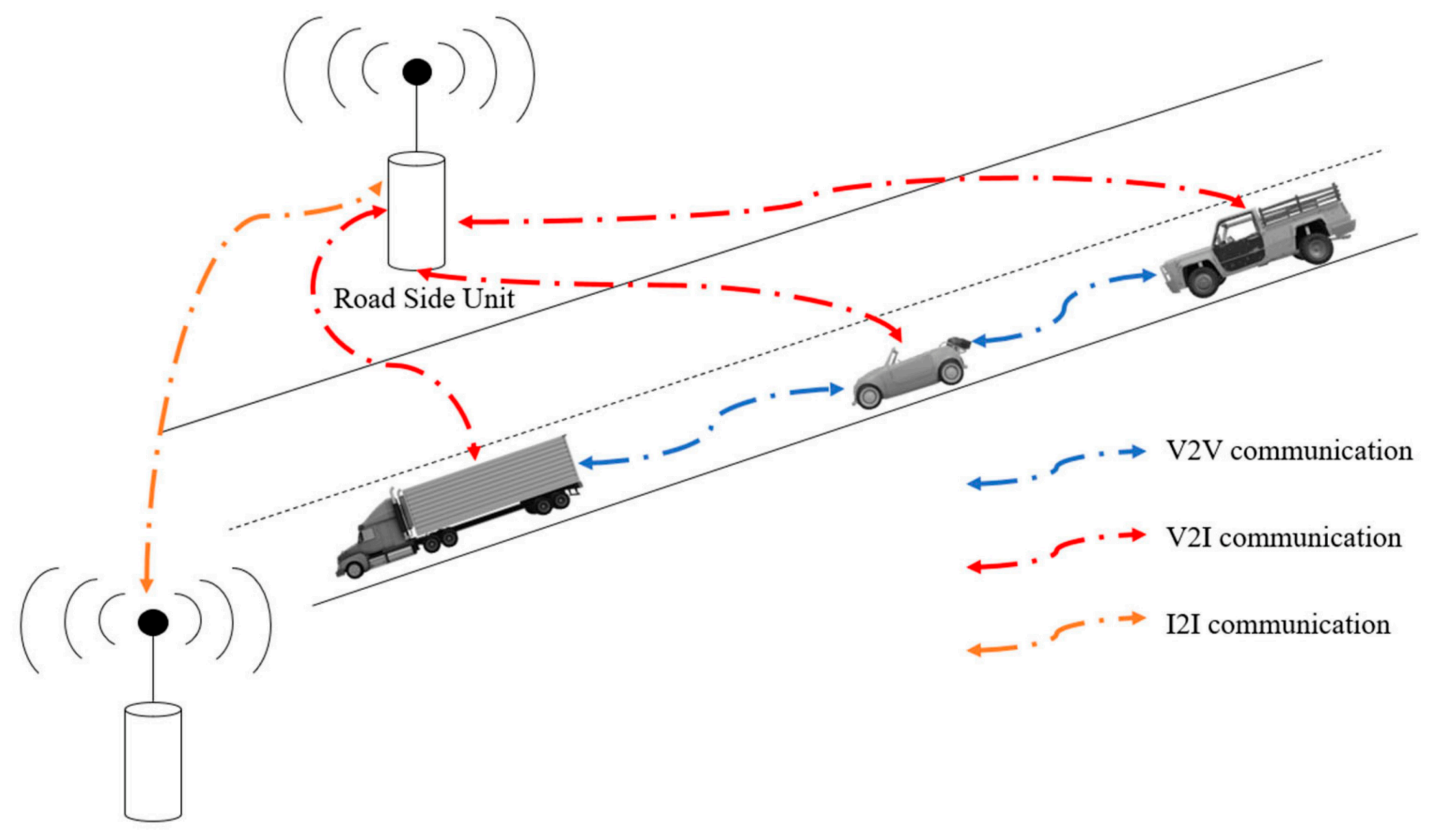
Main communication modes within V2X ecosystems, including Vehicle-to-Vehicle (V2V), Vehicle-to-Infrastructure (V2I), and Infrastructure-to-Infrastructure (I2I) communication [2].

Figure 6 illustrates the fundamental communication relationships within a V2X ecosystem. Vehicle-to-Vehicle (V2V) communication enables direct information exchange between neighboring vehicles, allowing the sharing of data related to position, speed, acceleration, and driving intentions. Vehicle-to-Infrastructure (V2I) communication facilitates interactions between vehicles and roadside units (RSUs), providing access to traffic management services, road condition information, and safety warnings. In addition, Infrastructure-to-Infrastructure (I2I) communication enables coordination between distributed roadside units and traffic management systems, supporting broader transportation network optimization. The integration of these communication modes creates a cooperative transportation environment in which information can be rapidly disseminated among multiple participants, thereby improving situational awareness, traffic efficiency, and road safety.

The V2X communication paradigm encompasses several communication categories, each designed to address specific operational requirements. Vehicle-to-Vehicle (V2V) communication enables direct information exchange between nearby vehicles, allowing the sharing of position, speed, acceleration, braking status, and driving intentions to improve situational awareness and support safer vehicle operation [14]. Vehicle-to-Infrastructure (V2I) communication facilitates interaction between vehicles and roadside infrastructure, providing information related to traffic conditions, signal timing, speed limits, road construction activities, and hazardous situations [13,14]. Vehicle-to-Network (V2N) communication connects vehicles to cloud platforms, communication networks, and external services, enabling access to navigation systems, software updates, predictive maintenance platforms, and cloud-based analytics [13]. Vehicle-to-Pedestrian (V2P) communication focuses on protecting vulnerable road users by enabling communication between vehicles and mobile devices carried by pedestrians or cyclists. Dadashev and Török [53] demonstrated the SmartDENM system, which combines machine vision and V2X communication to improve pedestrian safety in complex traffic environments.

The evolution of communication technologies has expanded the range of platforms available for V2X applications. Cellular V2X (C-V2X) has emerged as one of the dominant communication approaches for connected mobility. Du et al. [54] investigated the spatial characteristics of interference in C-V2X communication systems, highlighting the importance of communication planning and interference management in dense transportation environments. Alternative communication technologies may also complement conventional V2X solutions under specific operating conditions. Haque et al. [55] proposed a LoRa-based V2X architecture capable of supporting long-range connectivity with low energy consumption, while Jia et al. [56] developed a high-data-rate V2X communication system integrating Multiple-Input Multiple-Output Visible Light Communication (MIMO VLC) and radar technologies. These studies illustrate the growing technological diversity of future V2X ecosystems and the increasing convergence of communication and sensing functionalities.

The continuous development of V2X communication technologies has transformed transportation systems from isolated entities into highly connected cooperative networks. Modern V2X architectures increasingly combine multiple communication technologies, including 5G, C-V2X, DSRC, LoRa, optical communication, and edge computing infrastructures. Khalid et al. [13] proposed an intelligent technology selection algorithm capable of dynamically choosing among different communication technologies according to operational requirements and network conditions. Such hybrid approaches are expected to play a critical role in future transportation systems where no single communication technology can independently satisfy all application requirements.

Despite the diversity and capabilities of modern V2X technologies, several challenges remain. Interoperability among heterogeneous communication systems, spectrum management, communication reliability in dense traffic environments, cybersecurity protection, and large-scale infrastructure deployment continue to represent important barriers to widespread adoption. Addressing these challenges will be essential for achieving fully connected and cooperative mobility ecosystems.

In essence, V2X communication provides the technological foundation for cooperative mobility ecosystems by enabling real-time information exchange among vehicles, infrastructure, pedestrians, and external services. The diversity of available communication modes and technologies allows transportation systems to support a broad range of safety, efficiency, and automation applications while creating the necessary communication framework for future connected and autonomous mobility.

### 4.2. Applications of V2X Communication

The practical significance of Vehicle-to-Everything communication lies in its ability to support a wide range of applications that improve transportation safety, efficiency, automation, and user experience. By enabling continuous information exchange among vehicles, infrastructure, communication networks, and other road users, V2X communication transforms traditional transportation systems into cooperative and intelligent mobility ecosystems. These capabilities are becoming increasingly important as modern vehicles generate larger volumes of data and autonomous driving technologies continue to advance [13,14].

**Figure 7:**
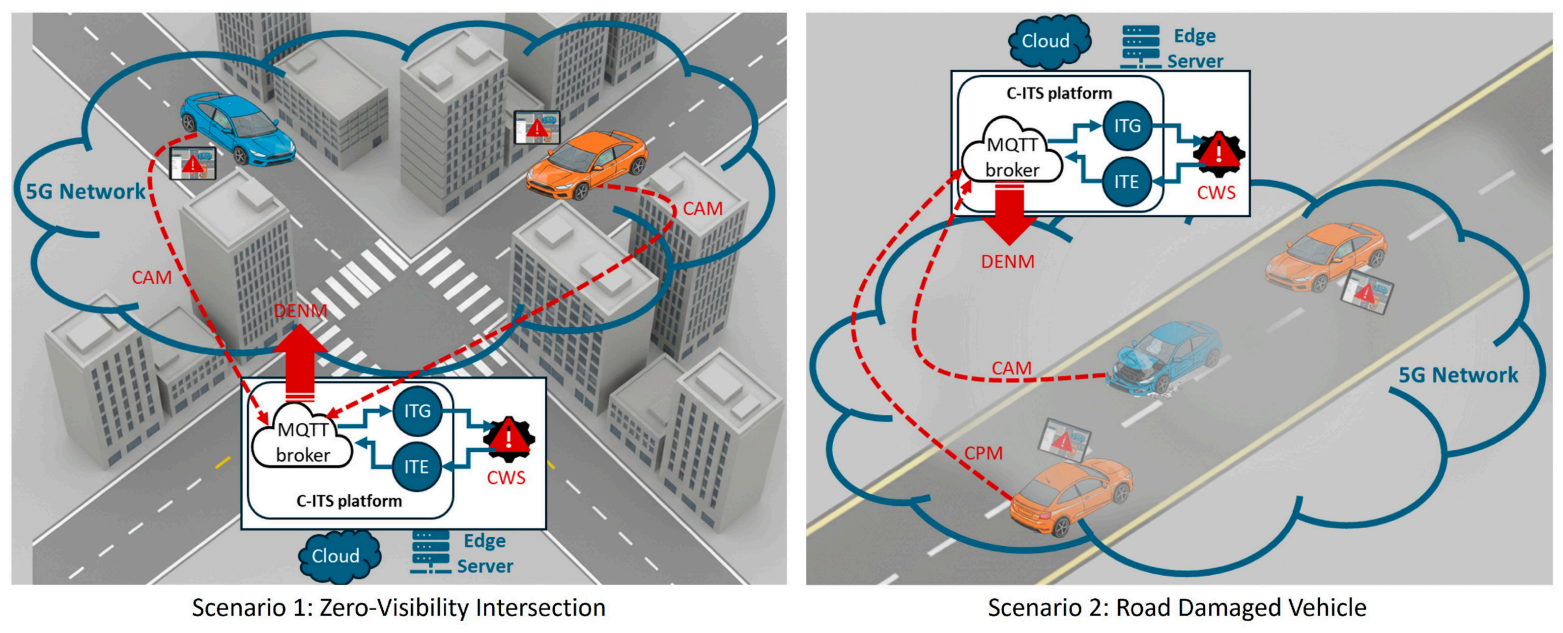
Examples of V2X-enabled safety applications in connected transportation systems, including zero-visibility intersection assistance and damaged vehicle warning scenarios [6].

Figure 7 presents two representative V2X application scenarios supported by 5G-enabled Cooperative Intelligent Transport Systems (C-ITS). In the first scenario, communication between vehicles, roadside infrastructure, and cloud-based services enables the detection of potential hazards at a zero-visibility intersection, where direct line-of-sight observation is limited by surrounding buildings. By exchanging Cooperative Awareness Messages (CAM) and Decentralized Environmental Notification Messages (DENM), vehicles can receive early warnings about approaching traffic and potential collision risks. The second scenario demonstrates the detection and dissemination of information regarding a disabled or damaged vehicle. In this case, hazard notifications are transmitted through the communication infrastructure to nearby vehicles, allowing drivers or autonomous systems to adapt their behavior and avoid dangerous situations. These examples illustrate how V2X communication extends situational awareness beyond the capabilities of onboard sensors and contributes to improved road safety, traffic efficiency, and cooperative mobility services.

One of the most important application domains of V2X communication is transportation safety. Through real-time information exchange, vehicles can detect hazardous situations beyond the range of onboard sensors and respond proactively to potential risks. V2X systems support collision avoidance, emergency braking warnings, intersection safety management, and cooperative awareness applications. Jung et al. [14] demonstrated a V2X-assisted autonomous driving framework in which communication between vehicles and infrastructure improves situational awareness and supports safer vehicle operation. The protection of vulnerable road users represents an important extension of these capabilities. Dadashev and Török [53] proposed the SmartDENM system, which combines machine vision and V2X communication to improve pedestrian safety and provide timely warnings to both drivers and pedestrians in complex traffic environments.

**Figure 8:**
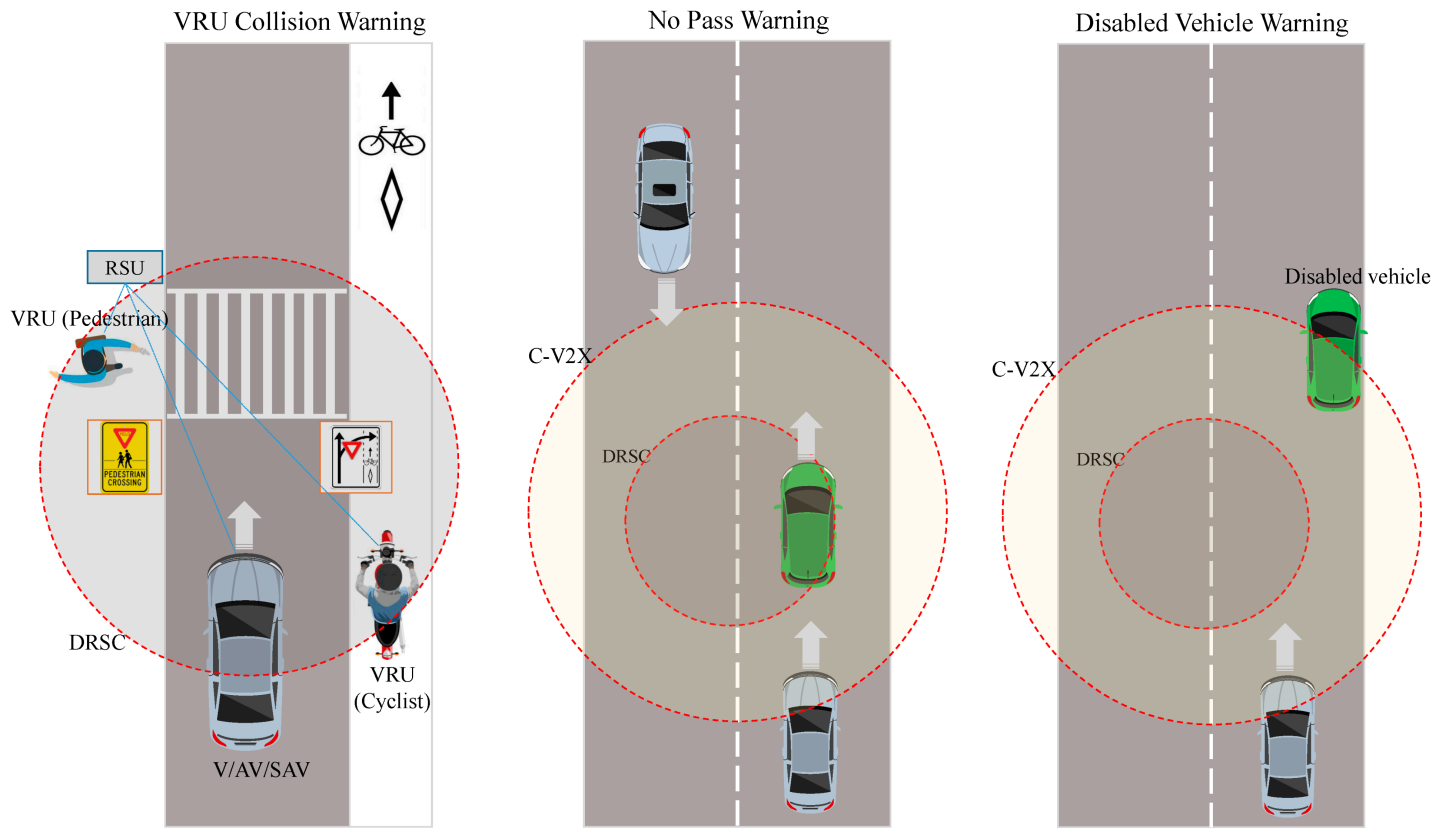
Examples of V2X-based safety applications, including vulnerable road user protection, no-pass warning, and disabled vehicle warning scenarios [1]. The shaded circles represent the DSRC communication range, the dashed red circles represent the C-V2X communication range, and the white arrows indicate the direction of travel.

Figure 8 illustrates several representative safety applications enabled by V2X communication technologies. The first scenario focuses on Vulnerable Road User (VRU) protection, where communication between vehicles, roadside infrastructure, and pedestrians or cyclists allows the early detection of potential collision risks. By transmitting warning messages beyond the direct line of sight of the driver, the system can significantly improve safety in urban environments and at pedestrian crossings. The second scenario demonstrates a no-pass warning application, where vehicles exchange information regarding traffic conditions and vehicle positions to prevent unsafe overtaking maneuvers. Such cooperative awareness mechanisms help reduce the likelihood of head-on collisions and other dangerous traffic situations. The third scenario presents a disabled vehicle warning system, in which information about a stationary or malfunctioning vehicle is transmitted to approaching vehicles, allowing drivers or autonomous systems to take preventive actions. Together, these applications demonstrate how V2X communication extends environmental awareness beyond the capabilities of onboard sensors and contributes to safer and more proactive transportation systems.

V2X communication also plays a central role in supporting connected and autonomous vehicle operations. Autonomous vehicles require continuous access to information regarding surrounding traffic conditions, vehicle intentions, infrastructure status, and environmental changes. Communication-based situational awareness complements onboard perception systems and improves decision-making accuracy. Recent developments have further integrated artificial intelligence into V2X environments. Li et al. [57] proposed a reinforcement learning-based resource allocation scheme for NR-V2X communication, demonstrating how intelligent communication management can optimize both sensing and communication performance. The integration of V2X communication with edge computing infrastructures further enhances these capabilities by enabling low-latency data processing and rapid decision-making for safety-critical mobility services [58].

Traffic management and transportation efficiency constitute another major application domain. Through the exchange of real-time traffic information, vehicles can receive route recommendations, congestion warnings, and infrastructure status updates. Wang et al. [58] developed a short-term traffic state prediction framework based on mobile edge computing and V2X communication, illustrating how distributed computing resources can improve traffic forecasting accuracy and support intelligent transportation management. Such approaches contribute to reducing congestion, improving traffic flow, decreasing travel times, and optimizing transportation network performance.

Emerging V2X applications increasingly incorporate advanced digital technologies such as blockchain and adaptive communication management. Zrikem et al. [59] introduced the concept of Vehicle-to-Blockchain (V2B) communication, demonstrating how decentralized architectures can enhance trust management, authentication, and secure information exchange within connected transportation systems. Similarly, Khalid et al. [13] proposed an intelligent technology selection algorithm capable of dynamically choosing among 5G, C-V2X PC5, and DSRC communication technologies according to operational requirements and network conditions. These approaches illustrate how future transportation systems may utilize multiple communication platforms simultaneously to optimize service performance.

Another emerging application area involves simulation and virtual testing environments designed to support the development and validation of connected and autonomous vehicles. BaniSalman et al. [60] proposed VRDeepSafety, a virtual reality simulation platform integrating V2X communication for accident prediction and safety evaluation. Such environments allow researchers and developers to assess communication strategies, traffic scenarios, and vehicle behavior under controlled conditions before deployment in real-world transportation systems.

Despite the growing diversity of V2X applications, their successful large-scale deployment remains dependent on communication reliability, infrastructure availability, interoperability among heterogeneous systems, cybersecurity protection, and regulatory standardization. Addressing these challenges will be essential for realizing the full potential of cooperative and intelligent transportation ecosystems.

On balance, V2X communication has evolved from a simple communication concept into a critical enabling technology for intelligent transportation systems. Its applications now extend beyond basic vehicle communication to include autonomous driving, pedestrian protection, traffic management, edge computing, blockchain integration, and virtual testing environments. As communication technologies continue to advance, V2X communication is expected to play an increasingly central role in the development of safer, more efficient, and more autonomous mobility ecosystems.

At the same time, a critical reading of the V2X literature reveals important limitations. The majority of reported results originate from simulations or small-scale pilots involving at most tens of vehicles, and evaluation methodologies differ so widely—in traffic density, channel models, message rates, and performance metrics—that quantitative results are rarely comparable across studies. Evidence on communication performance under high channel load is partly contradictory: while several works report that congestion control mechanisms preserve acceptable latency at high vehicle densities, others document substantial packet loss precisely in the dense urban scenarios where cooperative safety applications would be most valuable. Furthermore, most experimental deployments assume homogeneous technology penetration, whereas realistic mixed fleets of DSRC-equipped, C-V2X-equipped, and non-equipped vehicles remain insufficiently studied. These methodological limitations should be taken into account when generalizing the reported benefits of V2X systems [60].

## 5. Emerging Trends in Connected Mobility

The rapid evolution of connected mobility is transforming vehicles from isolated transportation units into intelligent, software-driven platforms capable of continuously interacting with their surrounding environment. Advances in communication technologies, artificial intelligence, cloud computing, edge computing, and data analytics have accelerated the development of new mobility paradigms that extend beyond conventional vehicle operation. Among the most significant trends shaping the future of transportation are Software-Defined Vehicles (SDVs), artificial intelligence-enabled mobility systems, and digital twin technologies.

Unlike traditional automotive architectures that rely heavily on dedicated hardware functionalities, future mobility platforms increasingly depend on software-centric approaches capable of supporting continuous updates, adaptive functionality, and intelligent decision-making. Simultaneously, the growing volume of telemetry and operational data creates new opportunities for artificial intelligence to enhance autonomous driving, predictive maintenance, traffic optimization, and personalized mobility services. Digital twin technologies further extend these capabilities by enabling the creation of dynamic virtual representations of physical assets that continuously evolve based on real-time operational information. Together, these technologies are expected to redefine how vehicles are designed, operated, maintained, and integrated within broader transportation ecosystems.

**Figure 9:**
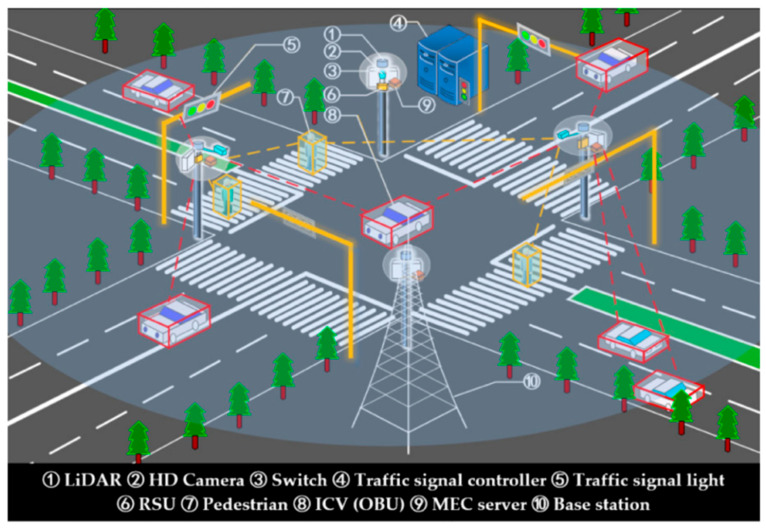
Intelligent connected mobility ecosystem integrating vehicles, roadside infrastructure, edge computing, and communication networks within a smart transportation environment [58]. Dashed lines represent wireless communication links between the infrastructure elements, vehicles, and the MEC server; solid yellow lines represent the wired connections of the roadside infrastructure; and thin white leader lines connect the numbered labels with the corresponding components.

Figure 9 illustrates an intelligent connected mobility ecosystem integrating vehicles, roadside infrastructure, communication networks, and edge computing resources within a smart intersection environment. The architecture combines multiple sensing and communication technologies, including LiDAR systems, HD cameras, roadside units (RSUs), traffic signal controllers, Multi-access Edge Computing (MEC) servers, and cellular base stations. Real-time data collected from vehicles, pedestrians, and infrastructure elements are processed and exchanged through edge and cloud platforms to support traffic management, cooperative perception, and autonomous driving applications. Such integrated environments represent the foundation of future software-defined and data-driven transportation systems, where artificial intelligence, edge computing, and digital twins continuously analyze operational conditions and optimize transportation performance. The convergence of physical infrastructure and digital services enables more efficient traffic control, enhanced situational awareness, improved safety, and the seamless integration of connected and autonomous vehicles into urban mobility ecosystems.

Despite their transformative potential, emerging mobility technologies also introduce significant challenges related to software complexity, large-scale data management, cybersecurity protection, system validation, and regulatory compliance. Addressing these challenges will be essential for ensuring the safe and reliable deployment of future connected and autonomous transportation systems.

The following sections examine the principles, applications, benefits, and challenges associated with Software-Defined Vehicles, artificial intelligence-enabled mobility systems, and digital twin technologies, highlighting their growing role within next-generation connected mobility ecosystems.

### 5.1. Software-Defined Vehicles and Artificial Intelligence

Software-Defined Vehicles (SDVs) represent a fundamental shift in automotive system architecture. In traditional vehicles, most functionalities are tightly coupled with dedicated hardware components and electronic control units. In contrast, SDVs increasingly rely on centralized computing platforms where software becomes the primary mechanism for implementing and updating vehicle functions. This approach allows manufacturers to continuously improve vehicle capabilities throughout their operational lifecycle without requiring physical hardware modifications [61].

One of the defining characteristics of SDVs is the ability to deploy Over-the-Air (OTA) software updates. OTA technologies enable manufacturers to remotely distribute software patches, cybersecurity updates, performance improvements, and entirely new functionalities directly to vehicles operating in the field. Such capabilities significantly enhance vehicle flexibility and reduce maintenance requirements. However, the increasing dependence on remote software deployment also introduces new security challenges. Shin and Jeon [62] proposed the MQTree protocol, which combines MQTT communication and Merkle Tree verification mechanisms to improve the security and integrity of OTA update processes, highlighting the growing importance of secure software management within future automotive ecosystems.

The development of SDVs is closely associated with the broader adoption of artificial intelligence within connected mobility systems. Modern vehicles generate large volumes of telemetry data originating from sensors, communication networks, driver interactions, and vehicle subsystems. Artificial intelligence provides the computational capability required to analyze these datasets and extract actionable information. Garikapati and Shetiya [61] emphasized the central role of AI technologies in the evolution of autonomous vehicles, highlighting their contributions to perception, decision-making, route planning, vehicle control, and predictive maintenance. Machine learning algorithms can continuously analyze vehicle-generated data to identify operational anomalies, forecast potential component failures, and support proactive maintenance strategies, thereby improving vehicle reliability, reducing operational costs, and enhancing transportation safety.

The software-centric nature of modern vehicles has also increased the importance of cybersecurity, digital forensics, and operational transparency. Wang et al. [63] proposed an intrusion detection system based on the Swin Transformer architecture for hybrid automotive in-vehicle networks, demonstrating how artificial intelligence can improve the detection of malicious activities while minimizing false-positive alerts. Similarly, Lee et al. [64] investigated Tesla vehicle log data from a digital forensics perspective, illustrating the growing importance of software-generated records for diagnostics, accident investigations, system validation, driver behavior analysis, and cybersecurity monitoring.

Despite their considerable advantages, Software-Defined Vehicles and AI-enabled mobility systems introduce significant challenges related to software complexity, cybersecurity protection, validation of machine learning models, data privacy, and regulatory compliance. As vehicle functionality increasingly shifts from hardware to software, ensuring system reliability, security, and transparency becomes essential for the safe deployment of future connected and autonomous transportation systems.

In summary, Software-Defined Vehicles and artificial intelligence represent a major transformation in automotive engineering. By shifting functionality from hardware to software and incorporating intelligent data analytics into vehicle operation, these technologies create adaptive, continuously evolving mobility platforms capable of supporting autonomous driving, predictive maintenance, advanced cybersecurity, and personalized transportation services.

### 5.2. Digital Twins in Automotive Systems

The growing availability of real-time telemetry data, advanced communication infrastructures, and cloud-based computing resources has accelerated the adoption of digital twin technologies within connected mobility ecosystems. A digital twin can be defined as a virtual representation of a physical asset, process, or system that continuously evolves through synchronization with real-world data. By integrating telemetry information, communication networks, sensor measurements, and computational models, digital twins provide an environment for monitoring, simulation, optimization, and predictive decision-making [16,65,66].

**Figure 10:**
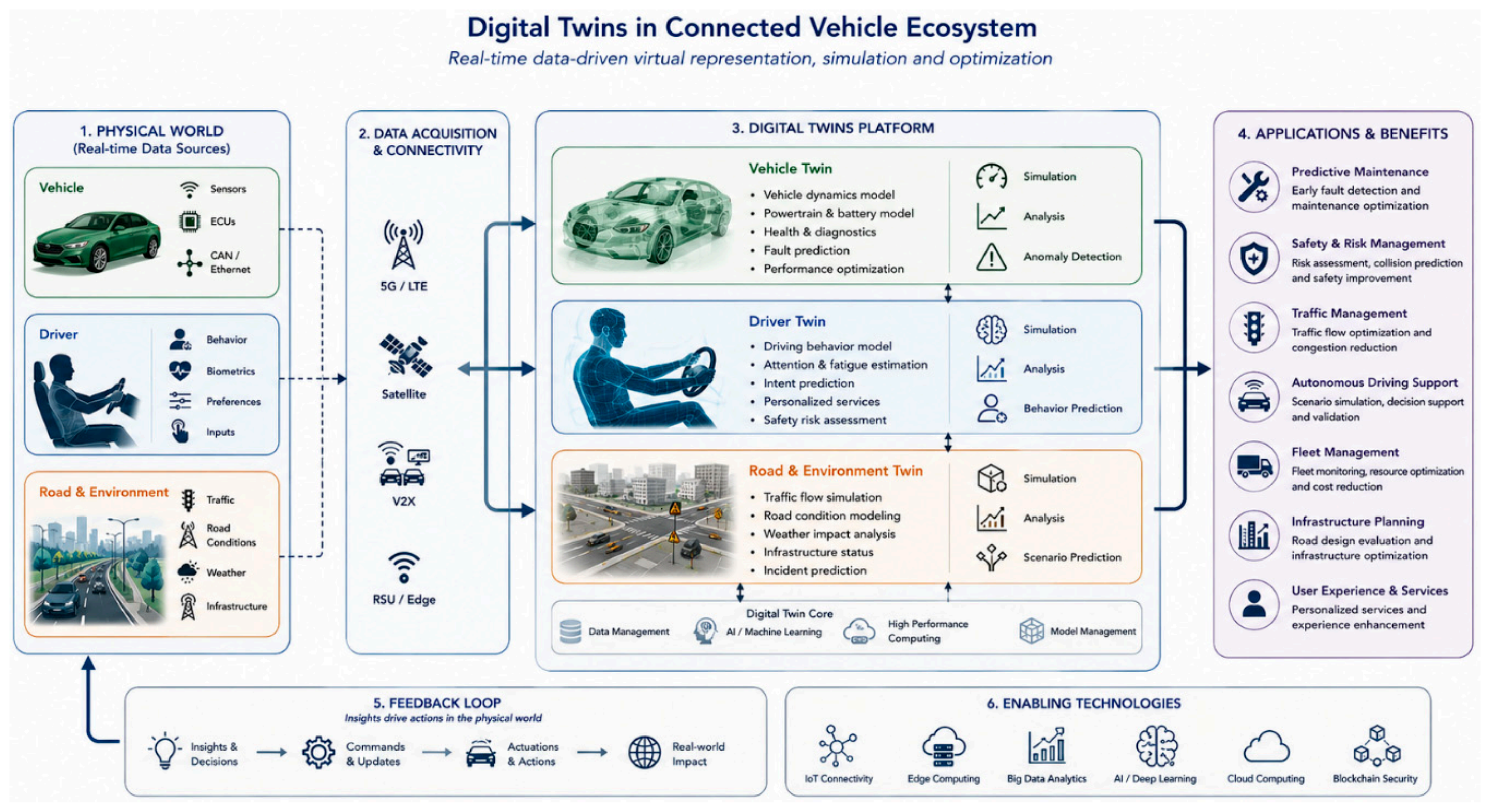
Multi-level digital twin architecture integrating vehicle, driver, and road environment twins in connected transportation systems.

The concept of digital twins has expanded significantly beyond its initial applications in industrial and manufacturing environments. Gunaratne et al. [65] presented a digital twins platform for digital manufacturing, demonstrating how virtual representations can support process monitoring, system optimization, and operational decision-making. These capabilities are increasingly being transferred to transportation systems, where continuously updated digital models can improve vehicle management, mobility services, and infrastructure planning. Figure 10 illustrates a multi-level digital twin architecture integrating vehicle, driver, and road environment twins within a connected mobility ecosystem. Real-time data collected through sensors, communication networks, and transportation infrastructure are continuously synchronized with virtual models, enabling simulation, prediction, optimization, and intelligent decision-making. The integration of artificial intelligence, edge computing, and cloud platforms further enhances the ability of digital twins to support predictive maintenance, traffic management, autonomous driving, and personalized mobility services.

**Figure 11:**
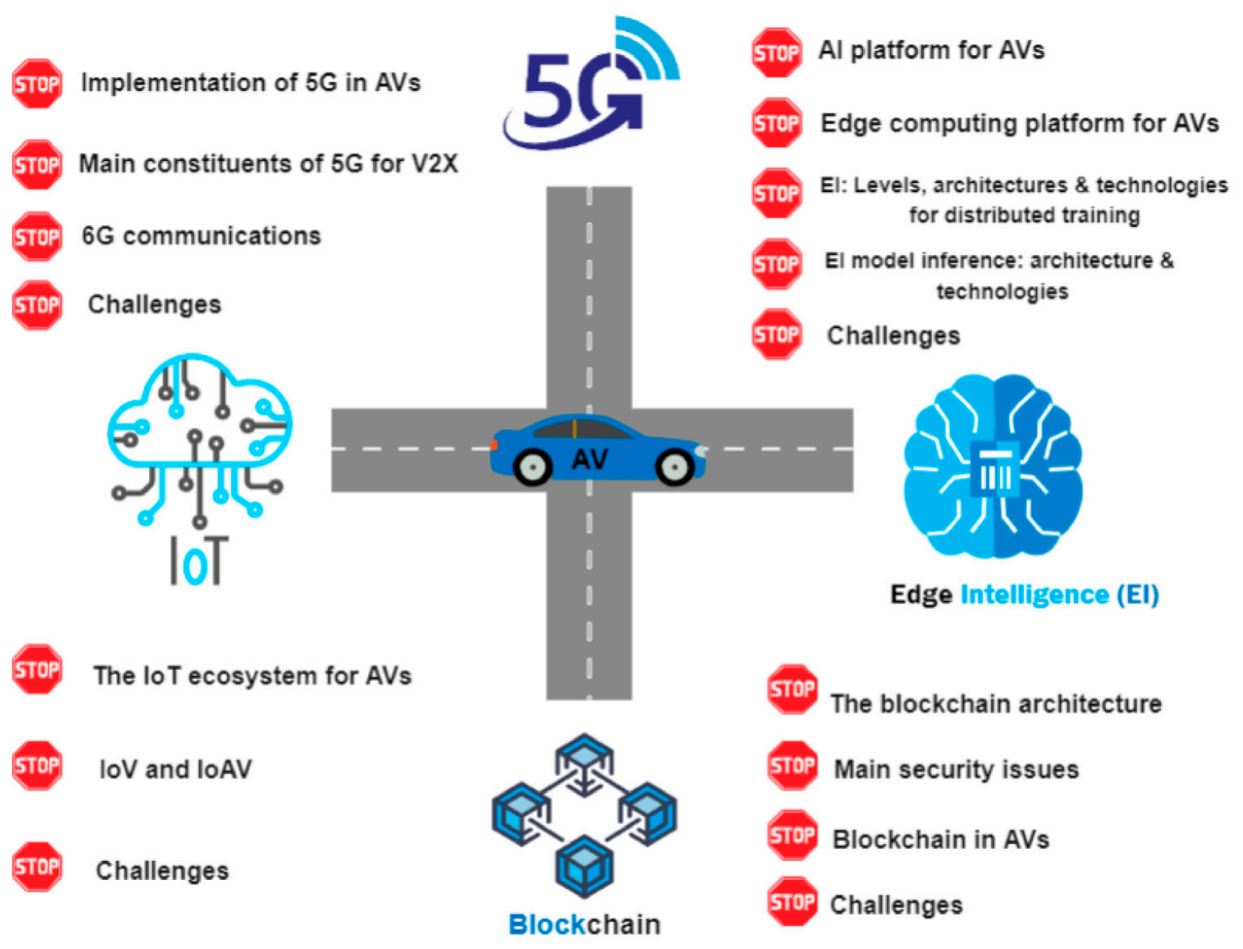
Integration of 5G communication, IoT, edge intelligence, artificial intelligence, and blockchain technologies in future connected and autonomous vehicle ecosystems [9].

Within the automotive domain, digital twins can be implemented at multiple levels. Kaya et al. [16] proposed a unified conceptual framework that incorporates vehicle, driver, and road digital twins into connected mobility ecosystems. Vehicle digital twins continuously replicate the operational state of a physical vehicle using telemetry data obtained from onboard sensors and communication systems. Driver digital twins model driving behavior and decision-making patterns, while road digital twins represent transportation infrastructure and traffic environments. The integration of these digital entities enables comprehensive situational awareness and supports intelligent transportation management. Figure 11 illustrates the technological ecosystem supporting next-generation connected and autonomous vehicles. Modern digital twin implementations do not operate as isolated virtual models but are increasingly integrated with complementary technologies, including 5G communication, Internet of Things (IoT) platforms, edge intelligence, artificial intelligence, and blockchain-based security frameworks. Through continuous data exchange enabled by 5G and IoT infrastructures, digital twins can receive real-time information from vehicles, drivers, and transportation infrastructure. Edge intelligence and AI algorithms process these large-scale datasets to support predictive analytics, traffic optimization, autonomous decision-making, and vehicle diagnostics. In parallel, blockchain technologies provide mechanisms for secure data sharing, authentication, and integrity verification within distributed mobility ecosystems. The convergence of these technologies creates a foundation for intelligent transportation systems capable of supporting real-time monitoring, simulation, and optimization of connected mobility services.

Digital twins are particularly valuable for predictive maintenance, lifecycle management, and operational optimization. By continuously monitoring vehicle condition and comparing real-world performance with virtual models, potential failures can be identified before they result in operational disruptions. This capability enables proactive maintenance planning, reduces downtime, improves reliability, and supports long-term vehicle performance evaluation throughout the operational lifecycle [16].

The development of autonomous vehicles has further increased interest in digital twin technologies. Autonomous driving systems require extensive testing under diverse operating conditions that may be difficult, costly, or unsafe to reproduce in real-world environments. Samak et al. [67] introduced the AutoDRIVE ecosystem, a comprehensive digital twin platform designed for autonomous driving research and education. Their framework enables the simulation of realistic driving scenarios, communication systems, and vehicle behaviors within a controlled virtual environment, accelerating algorithm development and validation while reducing the costs associated with physical testing.

Digital twins also provide opportunities for evaluating communication and cybersecurity mechanisms within connected vehicle ecosystems. Popa et al. [68] developed CarTwin, a digital twin of a real-world Controller Area Network (CAN), demonstrating how virtual environments can be used to analyze communication behavior, evaluate system performance, and investigate cybersecurity-related challenges without interfering with operational vehicles. This capability is becoming increasingly important as modern vehicles evolve into highly connected cyber-physical systems.

Future developments are expected to involve deeper integration of digital twins with artificial intelligence, edge computing, and large-scale mobility platforms. Perisic and Perisic [66] highlighted the growing role of intelligent analytics, real-time synchronization, and interconnected digital ecosystems in future digital twinning technologies. As computational capabilities continue to expand, digital twins are expected to evolve from isolated virtual models into collaborative environments capable of supporting autonomous decision-making, predictive analytics, intelligent transportation planning, and system-wide optimization. Future transportation ecosystems may consist of interconnected digital twins representing vehicles, drivers, infrastructure, communication networks, and entire urban environments, creating comprehensive digital mobility ecosystems [16,66].

Despite their considerable potential, digital twin systems face several challenges related to real-time data synchronization, model accuracy, computational requirements, interoperability, and cybersecurity protection. The effectiveness of digital twins is highly dependent on the quality and availability of telemetry data, making reliable communication infrastructures a critical prerequisite for their successful deployment.

Taken together, digital twins are expected to become one of the key technological foundations supporting the next generation of connected, autonomous, and data-driven mobility systems. Their ability to combine real-time monitoring, simulation, predictive analytics, and intelligent decision-making provides significant opportunities for improving transportation safety, operational efficiency, and mobility management.

A comparative assessment of the reviewed SDV, AI, and digital twin studies also exposes unresolved tensions. Proposed SDV architectures range from fully centralized high-performance computing designs to zonal and distributed approaches, and the literature provides little quantitative evidence about their relative reliability, cost, and update-management implications. In the AI domain, high-performing deep learning models conflict with the explainability and verifiability requirements of automotive safety standards, and most published models are trained on datasets that do not represent the full operational design domain of series-production vehicles. Digital twin research similarly faces a fidelity–latency trade-off: high-fidelity twins are computationally expensive and difficult to synchronize in real time over bandwidth-constrained vehicular links, while lightweight twins may be too coarse for safety-relevant decision support. The absence of standardized digital twin interfaces further prevents interoperability between platforms of different manufacturers.

The evolution of connected and autonomous vehicles is supported by the convergence of several advanced digital technologies. These technologies contribute to data acquisition, processing, decision-making, simulation, and secure information exchange within intelligent transportation systems. Their primary functions and representative applications are summarized in Table 4.

The technologies presented in Table 4 are highly interconnected and frequently operate together within modern connected mobility ecosystems. Their integration enables the development of intelligent, adaptive, and data-driven transportation services capable of supporting future autonomous mobility solutions.

## 6. Cybersecurity Challenges in Connected Mobility

The increasing digitalization of modern vehicles has significantly expanded the functionality of transportation systems while simultaneously introducing new cybersecurity challenges. Connected vehicles continuously exchange information with external communication networks, cloud platforms, roadside infrastructure, and other vehicles through various communication interfaces. While these capabilities enable advanced mobility services, autonomous driving functions, and real-time telemetry applications, they also increase the potential attack surface available to malicious actors. Consequently, cybersecurity has become a critical component of modern connected mobility ecosystems and a fundamental requirement for the safe deployment of intelligent transportation systems [25,41].

**Figure 12:**
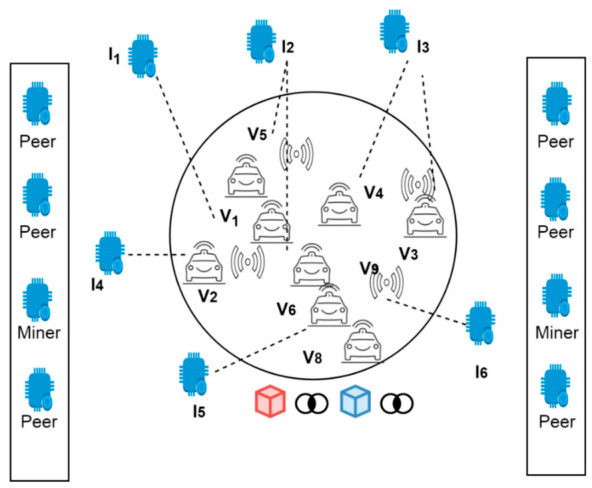
Blockchain-based communication architecture for connected vehicle networks and distributed trust management [27]. The red and blue cube symbols denote the two categories of blockchain blocks exchanged in the network (newly generated blocks and validated blocks appended to the distributed ledger, respectively), and the dashed lines indicate communication links between the infrastructure nodes (I1–I6) and the vehicles (V1–V9).

Unlike traditional vehicles, modern connected and software-defined vehicles operate as highly complex cyber-physical systems. Multiple electronic control units, communication gateways, wireless interfaces, cloud services, and over-the-air update mechanisms interact continuously throughout vehicle operation. Guan et al. [25] highlighted that intelligent connected vehicles face cybersecurity threats originating from both internal and external communication environments. Potential attacks may target communication channels, onboard networks, software platforms, sensors, or cloud infrastructures, potentially compromising vehicle functionality, data integrity, and user safety. Figure 12 presents a blockchain-enabled communication architecture designed for intelligent connected vehicles. In this framework, vehicles act as distributed network participants that exchange information through decentralized communication channels while interacting with external blockchain nodes. The use of blockchain technology provides a secure and transparent mechanism for data validation, authentication, and transaction verification without relying on a centralized authority. Such decentralized architectures can improve resistance against data tampering, spoofing attacks, and unauthorized access to vehicle-generated information. Furthermore, blockchain-based communication frameworks support trust management among connected vehicles by enabling secure sharing of telemetry, traffic, and safety-related data. As connected mobility ecosystems continue to expand, the integration of blockchain technologies is increasingly considered a promising approach for enhancing cybersecurity, data integrity, and communication reliability in intelligent transportation systems.

One of the most significant cybersecurity concerns involves unauthorized access to vehicle communication systems. Modern vehicles rely on internal communication networks to exchange information among subsystems responsible for vehicle control, diagnostics, and safety functions. If attackers gain access to these communication channels, they may manipulate transmitted data, disrupt vehicle operation, or interfere with safety-critical functions. These risks become increasingly important as vehicles evolve toward higher levels of connectivity, automation, and software dependency [25].

The growing adoption of Over-the-Air (OTA) software updates and 5G communication technologies has introduced additional cybersecurity challenges. OTA mechanisms enable manufacturers to remotely deploy software updates, security patches, and new functionalities throughout a vehicle’s operational lifecycle. Although these capabilities significantly improve maintainability and operational flexibility, compromised update mechanisms may provide opportunities for malicious software distribution and unauthorized system access. Shin and Jeon [62] proposed the MQTree protocol as a secure OTA update framework combining MQTT communication and Merkle Tree verification methods to improve software authenticity and update integrity. Similarly, Oh et al. [41] investigated security vulnerabilities associated with firmware over-the-air updates in 5G-enabled automotive environments, highlighting the need for robust protection mechanisms capable of securing highly connected transportation infrastructures.

Securing communication channels remains another major challenge. Modern transportation systems depend heavily on wireless communication technologies including 5G, V2X, satellite communication, Wi-Fi, and Bluetooth connections. Algarni and Thayananthan [42] proposed Software-Defined Networking (SDN)-based security solutions designed to improve the resilience of transportation services through adaptive threat detection, communication protection, and security policy enforcement.

As transportation ecosystems become increasingly distributed, advanced security approaches such as blockchain and artificial intelligence are attracting growing research interest. Rathee et al. [27] proposed a blockchain-based framework for securing connected and autonomous vehicles, while Zrikem et al. [59] introduced the concept of Vehicle-to-Blockchain (V2B) communication for integrating blockchain mechanisms into V2X and Internet of Things environments. In parallel, Wang et al. [63] demonstrated an intrusion detection system based on the Swin Transformer architecture for hybrid automotive networks, illustrating how machine learning techniques can improve attack detection accuracy while minimizing false-positive alerts. These approaches demonstrate the growing convergence of decentralized security architectures and intelligent threat detection mechanisms.

In addition to protecting vehicle systems, cybersecurity strategies must address the growing volume of sensitive data generated by connected vehicles. Modern telemetry platforms collect information regarding vehicle location, operational status, driver behavior, communication activities, and software operation. Lee et al. [64] demonstrated the forensic value of Tesla log data, illustrating how software-generated records can provide detailed insights into vehicle operation and user activities. While such information offers significant benefits for diagnostics and system analysis, it also raises important concerns regarding privacy protection, data ownership, and regulatory compliance.

Future connected mobility ecosystems will increasingly integrate autonomous vehicles, V2X communication, cloud services, artificial intelligence, digital twins, and software-defined architectures. Despite significant advances in cybersecurity technologies, the increasing complexity of connected mobility ecosystems continuously generates new attack surfaces that require proactive and adaptive security strategies. Effective cybersecurity frameworks must therefore combine secure communication protocols, robust authentication mechanisms, intelligent threat detection systems, privacy protection measures, and resilient software management practices.

Collectively, cybersecurity represents one of the most critical challenges facing the future of connected mobility. The successful deployment of intelligent transportation systems, autonomous vehicles, and large-scale telemetry services depends heavily on the ability to establish secure, reliable, and trustworthy communication environments. Continued research into communication security, blockchain technologies, artificial intelligence, intrusion detection systems, and software protection mechanisms will be essential for supporting the next generation of connected and autonomous transportation systems.

From a comparative perspective, the reviewed security approaches embody different and partly conflicting design philosophies. Standardized C-ITS credential management systems based on public-key infrastructures offer proven scalability and regulatory acceptance, but they rely on centralized trust anchors and introduce certificate-management overhead. Blockchain-based schemes, by contrast, promise decentralized trust and tamper-evident data sharing, yet the reviewed studies rarely quantify their latency and energy overheads, which conflict with the millisecond-level budgets of safety-critical V2X messaging. Regulatory instruments such as UNECE Regulations R155/R156 [69,70] and ISO/SAE 21434 [71] now impose mandatory cybersecurity engineering processes on manufacturers, but the literature provides limited evidence on how the proposed academic mechanisms can be certified under these frameworks. Finally, the anticipated advent of quantum computing threatens currently deployed public-key primitives, and post-quantum migration strategies for resource-constrained vehicular platforms remain largely unexplored.

The increasing connectivity of modern vehicles introduces numerous cybersecurity challenges that may affect communication reliability, data integrity, operational safety, and user privacy. The major cybersecurity threats identified in connected vehicle ecosystems and their corresponding mitigation strategies are summarized in Table 5.

As indicated in Table 5, cybersecurity in connected mobility requires a multi-layered protection approach involving secure communication protocols, authentication mechanisms, encryption techniques, intrusion detection systems, and blockchain-based trust frameworks.

## 7. Future Trends in Connected Mobility

The future of connected mobility will be shaped by the convergence of advanced communication technologies, artificial intelligence, software-defined architectures, and increasingly autonomous transportation systems. As vehicles become more connected, intelligent, and data-driven, future mobility ecosystems will extend beyond traditional transportation concepts and evolve into highly integrated cyber-physical environments. The continuous exchange of information among vehicles, infrastructure, cloud platforms, and digital services will create new opportunities for improving transportation safety, efficiency, sustainability, and user experience.

One of the most significant future developments is expected to be the emergence of sixth-generation (6G) communication networks. While 5G technologies currently provide the communication foundation for connected mobility, future transportation systems will generate substantially larger volumes of telemetry, sensor, and perception data. 6G networks are expected to offer significantly higher data rates, lower latency, improved reliability, and deeper integration of artificial intelligence into communication infrastructures. These capabilities will support advanced autonomous driving applications, real-time digital twins, large-scale cooperative perception systems, and intelligent transportation services operating on a global scale.

**Figure 13:**
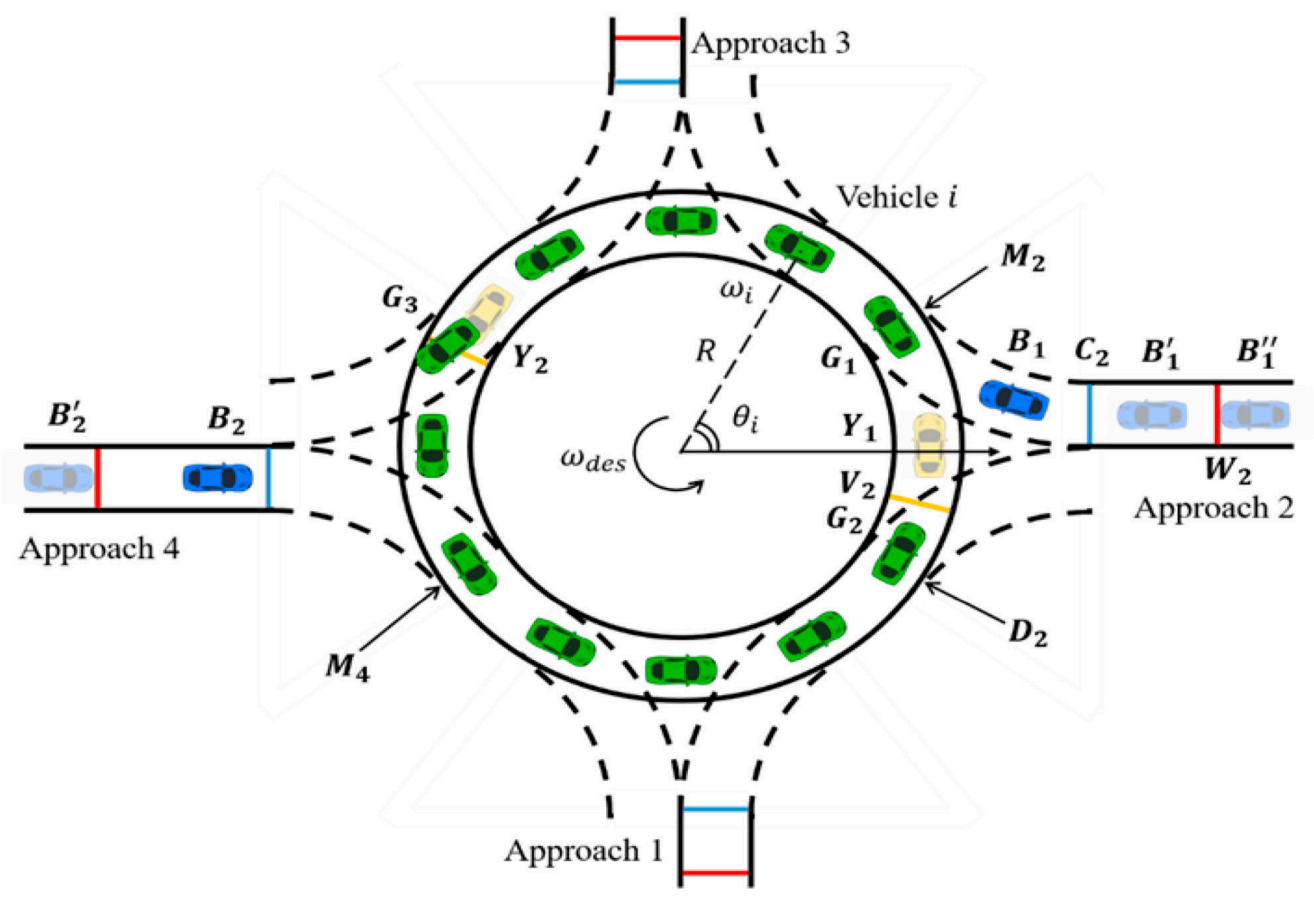
Cooperative autonomous driving scenario at a multilane roundabout based on V2X information exchange and coordinated maneuver planning [29]. Dashed curves indicate the vehicle trajectories at the roundabout entries and exits, solid circles delimit the circulating lanes, and the short red, blue, and yellow segments mark the reference control lines at the roundabout approaches.

Figure 13 illustrates a cooperative autonomous driving scenario at a multilane roundabout, where connected vehicles exchange real-time information regarding position, velocity, trajectory, and intended maneuvers. Through V2X communication and intelligent decision-making algorithms, vehicles can coordinate merging, lane changes, and roundabout navigation while minimizing traffic conflicts and improving traffic flow efficiency. Such cooperative control strategies are expected to become increasingly important in future connected and autonomous transportation systems, where collective decision-making may enhance both safety and mobility performance.

Another major trend involves the expansion of Non-Terrestrial Networks (NTNs). Future communication ecosystems will increasingly integrate terrestrial cellular networks, satellite constellations, high-altitude platforms, and aerial communication systems into unified communication architectures. Such integration will provide seamless global connectivity and ensure continuous communication availability regardless of vehicle location. Hybrid communication infrastructures combining terrestrial and satellite resources are expected to become essential components of autonomous transportation systems, long-distance logistics operations, and smart city environments.

Artificial intelligence will continue to play a central role in the evolution of connected mobility. Future AI systems are expected to move beyond data analysis and increasingly support autonomous decision-making, predictive maintenance, traffic optimization, communication management, and cybersecurity monitoring. The integration of edge computing with artificial intelligence will further enable real-time analytics and localized decision-making capabilities directly within vehicles and roadside infrastructure.

Software-defined architectures and digital twin technologies are expected to become standard components of future transportation ecosystems. Vehicles will increasingly evolve into software-centric platforms capable of receiving continuous updates and adaptive functionality throughout their lifecycle. At the same time, interconnected digital twins representing vehicles, infrastructure, communication networks, and urban environments will support predictive analytics, intelligent transportation planning, and system-wide optimization through continuous synchronization with real-world data.

Autonomous driving technologies will continue to advance toward higher levels of automation. Future autonomous vehicles will increasingly depend on the integration of telemetry systems, V2X communication, artificial intelligence, and digital twin platforms to achieve reliable operation under complex real-world conditions. Cooperative autonomous driving, in which multiple vehicles coordinate their actions through continuous communication and shared situational awareness, is expected to become a key feature of next-generation transportation systems.

Cybersecurity will remain a critical priority as transportation systems become increasingly interconnected and software-dependent. Future cybersecurity frameworks are expected to combine artificial intelligence, blockchain technologies, adaptive security architectures, and advanced intrusion detection systems to address emerging threats and protect increasingly complex mobility environments.

Sustainability considerations will also influence the future development of connected mobility. Intelligent transportation systems supported by telemetry, V2X communication, and artificial intelligence can contribute to reducing energy consumption, optimizing traffic flow, and improving vehicle efficiency. The growing adoption of electric vehicles, smart charging infrastructures, and vehicle-to-grid technologies will further support the transition toward more sustainable transportation systems.

Despite significant technological progress, several challenges remain. Future transportation systems must address issues related to interoperability, standardization, regulatory compliance, privacy protection, large-scale infrastructure deployment, and cybersecurity resilience. Ensuring seamless cooperation among vehicles, communication networks, cloud platforms, and transportation authorities will require extensive collaboration between industry, academia, and policymakers.

Key research directions include the development of interoperable communication standards, explainable artificial intelligence for autonomous decision-making, scalable digital twin architectures, resilient cybersecurity frameworks, and seamless integration of terrestrial and non-terrestrial communication networks. Addressing these challenges will be essential for enabling the widespread deployment of future connected mobility technologies.

Ultimately, the future of connected mobility will be characterized by deeper integration between physical transportation systems and digital technologies. Advances in communication networks, artificial intelligence, software-defined architectures, digital twins, and autonomous driving will continue to transform the transportation sector, creating increasingly intelligent, adaptive, and interconnected mobility ecosystems. These developments are expected to play a crucial role in shaping the next generation of transportation systems and supporting safer, more efficient, and more sustainable mobility solutions worldwide.

## 8. Discussion

The reviewed literature demonstrates that connected mobility is evolving toward highly integrated cyber-physical transportation ecosystems in which communication, computation, sensing, and intelligent decision-making operate as interconnected components rather than independent technologies. Although automotive telemetry, connected vehicles, advanced communication infrastructures, V2X communication, Software-Defined Vehicles (SDVs), digital twins, and cybersecurity are often investigated separately, their practical deployment increasingly depends on their mutual integration. The collective findings suggest that future transportation systems will be defined not only by individual technological advances but also by the ability to create interoperable and cooperative mobility environments.

A key observation emerging from the reviewed studies is the growing importance of vehicle-generated data as a strategic resource for intelligent mobility services. Galinski et al. [4] demonstrated that modern telemetry platforms can generate comprehensive datasets describing real-world driving conditions through the integration of cameras, GNSS receivers, and communication modules. Similarly, de Lima et al. [5] showed that telemetry data can support driver behavior classification and intelligent mobility analytics. The studies conducted by Hasibuan and Ma’arif [22] and Farroni et al. [23] further illustrated the increasing use of telemetry systems for real-time monitoring of electric vehicle batteries and tire performance. Collectively, these findings indicate that telemetry data have evolved from a diagnostic resource into a fundamental enabler of data-driven transportation systems.

The reviewed literature also highlights the transition from isolated vehicles toward connected mobility ecosystems. Abdelkader et al. [1] described connected vehicles as a key foundation of future intelligent transportation systems, while Ahmed et al. [2] emphasized their potential to improve transportation safety, efficiency, and sustainability. Furthermore, Pipicelli et al. [3] characterized connected vehicles as cyber-physical systems that integrate communication, sensing, and computational capabilities. Although these studies consistently identify significant benefits associated with connectivity, they also reveal persistent challenges related to scalability, interoperability, and system complexity.

Communication infrastructure remains one of the most critical enabling technologies for connected mobility. Gohar and Nencioni [7] highlighted the importance of 5G communication for supporting intelligent transportation systems through low-latency and high-capacity connectivity. Similarly, Condoluci et al. [8] and Chatzoulis et al. [36] demonstrated the importance of 5G-enabled V2X architectures for cooperative mobility applications. However, several studies indicate that terrestrial communication infrastructures alone cannot guarantee ubiquitous connectivity. In response to this limitation, Völk et al. [11] and Uko et al. [12] investigated the integration of satellite communication into 5G ecosystems and demonstrated the potential of hybrid communication architectures. These findings suggest that future transportation systems will likely depend on heterogeneous communication environments combining terrestrial and non-terrestrial networks to ensure reliable connectivity under diverse operating conditions.

The reviewed V2X studies consistently demonstrate the importance of cooperative communication for improving transportation safety and operational efficiency. Jung et al. [14] experimentally validated a V2X-assisted autonomous driving framework and showed how communication can complement onboard sensing systems. Khalid et al. [13] further demonstrated that future transportation environments may benefit from adaptive communication strategies capable of dynamically selecting among multiple communication technologies. Moreover, Dadashev and Török [53] highlighted the role of V2X communication in protecting vulnerable road users, while Wang et al. [58] demonstrated its potential for traffic prediction and intelligent transportation management. Together, these studies indicate that V2X communication is evolving from a communication mechanism into a fundamental component of cooperative mobility ecosystems.

Another significant trend identified throughout the reviewed literature is the shift toward software-centric vehicle architectures. Garikapati and Shetiya [61] emphasized the growing role of artificial intelligence in enabling autonomous vehicle functionalities and supporting software-defined vehicle architectures. At the same time, Shin and Jeon [62] highlighted the increasing importance of secure over-the-air software update mechanisms as vehicle functionality becomes increasingly software-dependent. In addition, Lee et al. [64] demonstrated the value of software-generated vehicle data for forensic analysis and operational monitoring. These findings suggest that future vehicle innovation will be increasingly driven by software capabilities, intelligent analytics, and continuous lifecycle updates rather than purely hardware-based improvements.

The reviewed digital twin studies demonstrate a similar shift toward data-driven transportation management. Gunaratne et al. [65] showed how digital twins can support monitoring, optimization, and operational decision-making, while Kaya et al. [16] proposed a unified framework integrating vehicle, driver, and road digital twins within connected mobility environments. Furthermore, Samak et al. [67] demonstrated the value of digital twin ecosystems for autonomous driving research and validation, and Perisic [66] emphasized their growing role in predictive analytics and intelligent decision-making. The increasing integration of digital twins with artificial intelligence and real-time telemetry suggests that these technologies may become essential components of future transportation systems.

Despite the significant benefits identified throughout the reviewed literature, cybersecurity remains one of the most critical challenges facing connected mobility. Guan et al. [25] highlighted the expanding attack surface associated with intelligent connected vehicles, while Oh et al. [41] demonstrated vulnerabilities related to firmware over-the-air update mechanisms. To address these challenges, Algarni and Thayananthan [42] proposed adaptive security frameworks for future transportation services, and Wang et al. [63] demonstrated the effectiveness of artificial intelligence-based intrusion detection systems. Similarly, Rathee et al. [27] and Zrikem et al. [59] highlighted the potential of blockchain-based approaches for improving trust, authentication, and data integrity within connected mobility ecosystems. These studies collectively indicate that cybersecurity must evolve in parallel with advances in communication, automation, and software-defined mobility.

A recurring observation across the reviewed studies is that future mobility systems will not depend on a single enabling technology. Instead, their success will rely on the seamless integration of telemetry platforms, communication infrastructures, V2X communication, artificial intelligence, software-defined architectures, digital twins, and cybersecurity mechanisms. While substantial progress has been achieved within each individual domain, the interoperability and coordinated operation of these technologies remain insufficiently explored and represent important directions for future research.

Several research gaps can be identified, and it is instructive to consider why they persist. Interoperability among heterogeneous communication technologies remains unresolved largely for structural reasons: the relevant standards are developed by different bodies (3GPP, ETSI, IEEE, SAE) on different timelines, regional regulatory decisions have diverged, and there are few shared large-scale testbeds in which cross-technology solutions could be validated end to end. Similarly, the integration of terrestrial and non-terrestrial networks is technologically recent—normative NTN support only appeared in 3GPP Release 17—so automotive-grade NTN terminals, antennas, and roaming and billing arrangements are still immature, and no published study has yet demonstrated seamless 5G–LEO handover for vehicles at scale.

Scalability constitutes a second persistent gap. Most reviewed studies validate their proposals in simulation or in fleets of at most tens of vehicles, whereas production deployments must serve millions of vehicles generating terabyte-scale daily data volumes; the economic and architectural implications of this scale—network densification costs, backhaul dimensioning, and storage life-cycle management—are rarely quantified. Comparable barriers affect artificial intelligence and digital twins: AI-based functions cannot currently be certified under automotive safety standards because explainability and verification methods for deep models are immature, and digital twins lack standardized interfaces and real-time synchronization mechanisms that would allow twins from different vendors to interoperate. Cybersecurity research, in turn, tends to optimize security strength in isolation, while deployment requires simultaneously satisfying latency, energy, and certification constraints—a multi-objective problem that few studies address.

Deployment barriers beyond technology also explain the slow transition from pilots to practice: unresolved business models for roadside infrastructure investment, fragmented spectrum policy, privacy regulation constraining cross-border vehicle data flows, and the long renewal cycle of the vehicle fleet itself. From these observations, several open research questions follow: How can seamless session continuity be guaranteed across 5G and LEO segments for vehicles moving at highway speeds? Which minimum service levels can hybrid architectures contractually guarantee, and at what cost per vehicle? How can AI-driven telemetry analytics be made certifiable under ISO 26262 [73] and SOTIF? What are scalable architectures for synchronizing millions of vehicle digital twins? And how should post-quantum security be introduced into resource-constrained vehicular platforms without violating latency budgets?

To translate these open questions into an actionable agenda, Table 6 prioritizes the identified research gaps and assigns to each of them an indicative time horizon and concrete recommended actions. The prioritization reflects both the severity of each gap as a deployment barrier and the maturity of the available technological building blocks.

Taken together, the reviewed literature indicates that the future of connected mobility will be characterized by increasing technological convergence between physical transportation systems and digital infrastructures. The successful realization of next-generation transportation ecosystems will depend not only on advances within individual technological domains but also on the ability to integrate communication, computation, sensing, and security into coherent and reliable mobility platforms. Addressing the identified challenges and research gaps will be essential for enabling safer, more efficient, and more sustainable transportation systems in the coming decades.

Although significant advances have been achieved in individual technological domains, no single technology is capable of addressing all requirements of future connected mobility ecosystems. Automotive telemetry provides real-time situational awareness, whereas V2X communication extends environmental perception beyond onboard sensing capabilities. Similarly, 5G networks offer ultra-low latency communication but remain constrained by coverage limitations, while satellite communication provides global connectivity at the expense of increased latency. Consequently, future transportation systems are expected to rely on hybrid architectures that combine complementary communication technologies while integrating artificial intelligence, digital twins, and cybersecurity frameworks into unified mobility platforms.

The technologies discussed throughout this review contribute to different aspects of connected mobility and autonomous transportation systems. While each technology provides unique capabilities, they also present specific limitations and research challenges. A comparative overview of the principal technologies, their advantages, limitations, applications, and future research directions is presented in Table 7.

The comparison presented in Table 7 highlights that the future of intelligent transportation systems will depend on the effective integration of multiple complementary technologies. Addressing challenges related to interoperability, scalability, cybersecurity, real-time processing, and communication reliability remains essential for the successful deployment of next-generation connected mobility ecosystems.

A key observation emerging from the reviewed literature is that future connected mobility ecosystems will be characterized by technological convergence rather than technological replacement. Future transportation systems are unlikely to rely on individual solutions operating independently. Instead, the integration of telemetry platforms, V2X communication, 5G and satellite networks, artificial intelligence, digital twins, edge computing, and cybersecurity mechanisms will form interconnected mobility ecosystems capable of supporting autonomous, intelligent, and data-driven transportation services.

**Figure 14:**
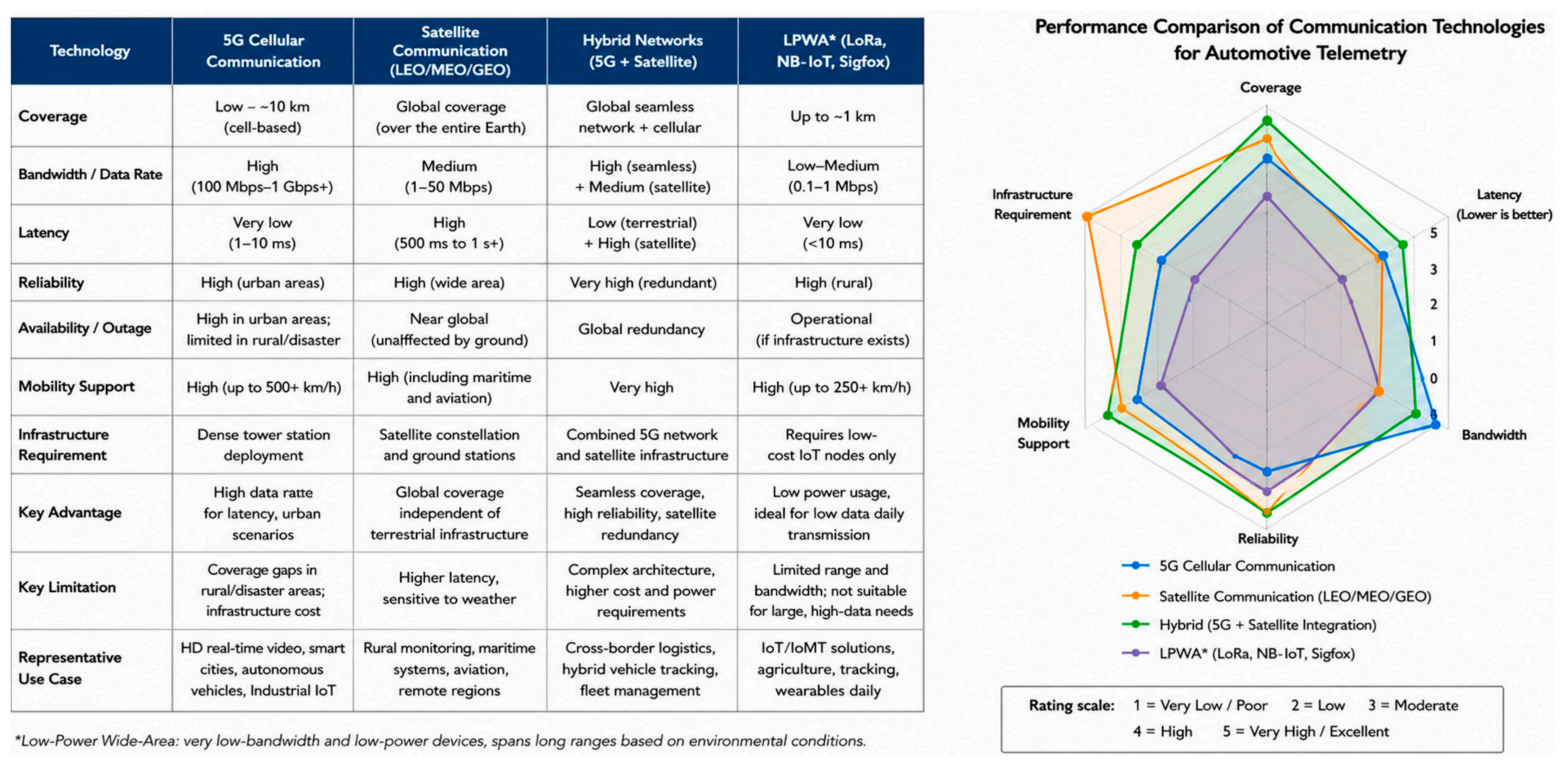
Comparative performance analysis of 5G, satellite, hybrid 5G–satellite, and DSRC/C-V2X communication technologies for automotive telemetry applications.

Figure 14 provides a comparative overview of the communication technologies discussed throughout this review. The comparison highlights the trade-offs between coverage, latency, bandwidth, reliability, mobility support, and infrastructure dependence for 5G, satellite communication, hybrid 5G–satellite architectures, and DSRC/C-V2X solutions. The results demonstrate that future connected mobility ecosystems will likely rely on hybrid communication approaches that combine the complementary strengths of multiple communication technologies.

**Figure 15:**
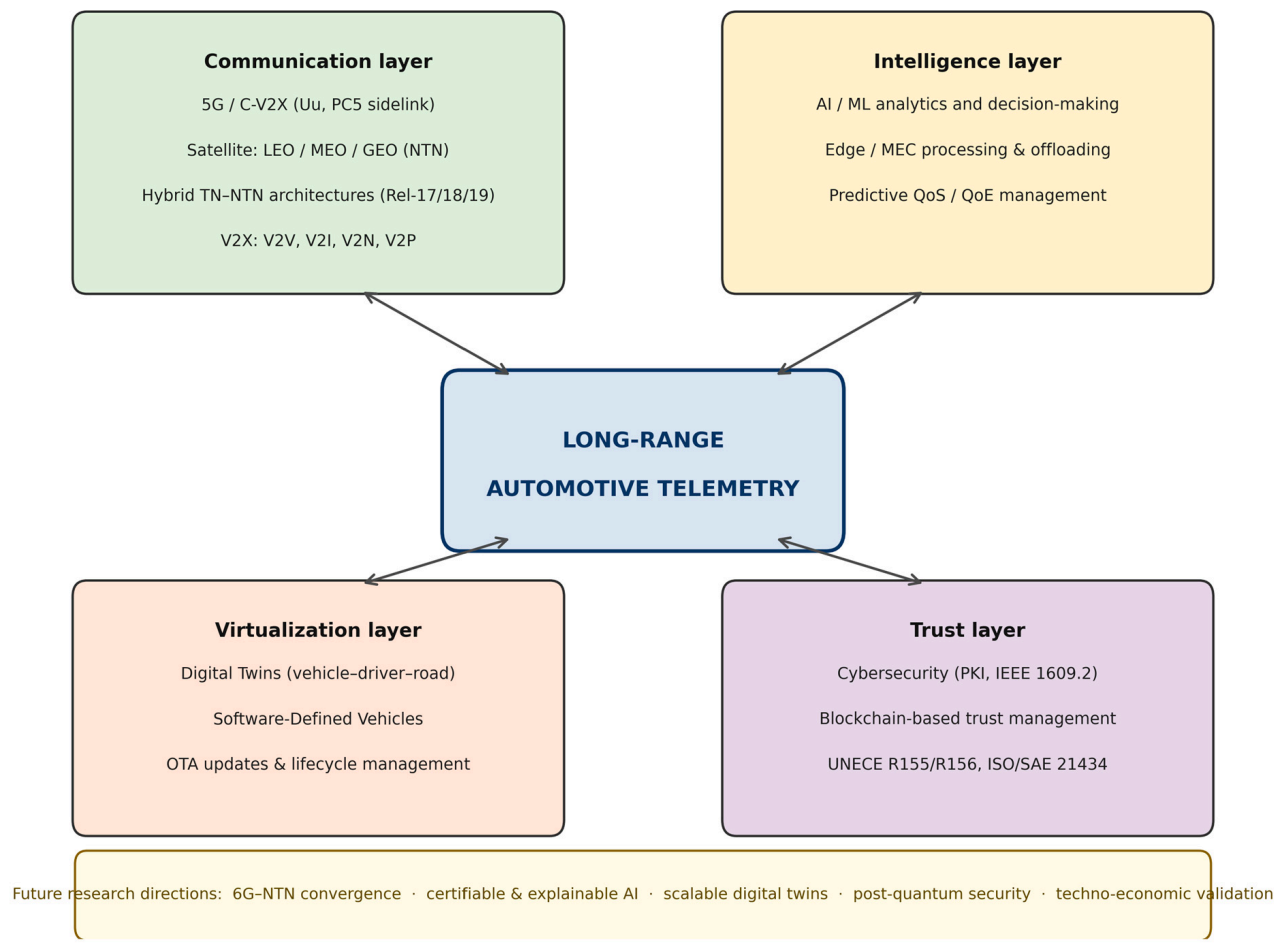
Taxonomy of the technological domains reviewed in this study and their mutual relationships.

To consolidate the relationships among the reviewed domains, Figure 15 presents a taxonomy that links long-range automotive telemetry to its enabling communication technologies, intelligence and virtualization layers, cross-cutting trust and cybersecurity mechanisms, and the corresponding future research directions identified in this review.

## 9. Conclusions

The rapid advancement of digital technologies is fundamentally transforming the automotive industry and reshaping the future of transportation systems. This review examined the major technological developments driving connected mobility, including automotive telemetry, connected vehicle technologies, advanced communication infrastructures, Vehicle-to-Everything (V2X) communication, Software-Defined Vehicles (SDVs), digital twins, artificial intelligence, and cybersecurity solutions. Collectively, these technologies form the foundation of next-generation intelligent transportation ecosystems capable of supporting increasingly connected, autonomous, and data-driven mobility services.

The reviewed literature demonstrates that modern mobility systems are evolving from isolated transportation platforms toward highly integrated cyber-physical ecosystems. Automotive telemetry has become a critical source of operational data, connected vehicle technologies enable continuous information exchange, and advanced communication infrastructures provide the connectivity required for real-time mobility services. At the same time, V2X communication extends situational awareness beyond onboard sensing capabilities and supports cooperative transportation environments that improve safety, efficiency, and traffic management.

The findings further indicate that future transportation systems will increasingly rely on software-centric architectures, artificial intelligence, and digital twin technologies. Software-Defined Vehicles enable continuous functional evolution through software updates, while artificial intelligence supports autonomous decision-making, predictive maintenance, and intelligent transportation management. Digital twins complement these capabilities by providing virtual representations of physical transportation systems that support simulation, optimization, and predictive analytics. Together, these technologies create the technological foundation for future autonomous and intelligent mobility ecosystems.

The review also highlights the growing importance of hybrid communication infrastructures that combine terrestrial and non-terrestrial communication technologies. While 5G networks provide low-latency and high-bandwidth communication, satellite systems offer extensive geographical coverage and communication resilience. Their integration is expected to play a key role in supporting future connected and autonomous transportation services operating across diverse environments.

Despite the significant progress achieved in connected mobility, several challenges remain unresolved. Cybersecurity, interoperability, communication reliability, privacy protection, infrastructure deployment, regulatory compliance, and large-scale system integration continue to represent major barriers to widespread adoption. As transportation systems become increasingly interconnected and software-dependent, ensuring secure, trustworthy, and resilient operation will remain a critical requirement for future mobility ecosystems.

In summary, the reviewed literature indicates that the future of transportation will be characterized by the convergence of communication, sensing, computation, and intelligent decision-making technologies. The successful realization of next-generation connected mobility will depend not only on advances within individual technological domains but also on the seamless integration and interoperability of telemetry systems, communication networks, artificial intelligence, digital twins, software-defined architectures, and cybersecurity frameworks. Future research should therefore increasingly focus on holistic system integration, cross-domain interoperability, and scalable mobility architectures capable of supporting safe, efficient, and sustainable transportation systems worldwide.

Based on the analysis presented in this review, several concrete research recommendations can be formulated. First, hybrid terrestrial/non-terrestrial telemetry architectures should be validated experimentally on real vehicle fleets using commercially available Release-17/18 NTN chipsets, with particular attention to vertical handover performance between 5G and LEO segments at highway speeds. Second, the research community should establish common evaluation benchmarks—covering latency, reliability, scalability, and cost per vehicle—so that results obtained by different research groups become quantitatively comparable. Third, lightweight and certifiable security mechanisms compatible with ISO/SAE 21434 and UNECE R155/R156, including post-quantum primitives suitable for resource-constrained onboard platforms, should be prioritized over conceptually novel but practically unverifiable schemes. Fourth, standardized digital twin interfaces and federated learning approaches for privacy-preserving telemetry analytics deserve targeted standardization and research effort. Finally, techno-economic studies quantifying deployment costs and viable business models for hybrid communication infrastructures are urgently needed, since the absence of such analyses currently represents one of the main barriers between laboratory results and large-scale adoption.

Viewed as a roadmap, these recommendations can be phased over time. In the short term (approximately one to three years), the highest-priority actions are the experimental validation of 5G–LEO service continuity with commercial NTN chipsets and the establishment of common evaluation benchmarks, since both are prerequisites for the meaningful comparison of subsequent research results. In the medium term (approximately three to five years), effort should concentrate on certifiable AI-based telemetry analytics, standardized digital twin interfaces, and techno-economic models of hybrid communication infrastructures. In the longer term, post-quantum security migration and fully interoperable cross-border telemetry ecosystems represent the principal challenges. The prioritized gap analysis in Table 6 links each of these phases to concrete open questions and recommended actions and is intended to serve as a reference agenda for future research on long-range automotive telemetry.

## Figures and Tables

**Table 1:** Comparison of the scope of this review with representative recent survey and review articles.

Reference	Primary Focus	Telemetry	5G	Satellite/NTN	V2X	SDV/AI/DT	Cybersecurity
Gohar and Nencioni [7]	5G technologies for intelligent transportation systems	✗	✓	✗	(✓)	✗	(✓)
Kang et al. [10]	Security of satellite communication systems	✗	✗	✓	✗	✗	✓
Rinaldi et al. [17]	Non-terrestrial networks in 5G and beyond	✗	✓	✓	✗	✗	✗
Soto et al. [18]	C-V2X-based road-safety and traffic-efficiency applications	✗	(✓)	✗	✓	✗	✗
Ying et al. [19]	V2X communication security	✗	(✓)	✗	✓	✗	✓
**This review**	Long-range automotive telemetry in connected vehicles	✓	✓	✓	✓	✓	✓

✓ = covered in depth; (✓) = partially covered; ✗ = not covered.

**Table 2:** Quantitative comparison of satellite orbital regimes and high-altitude platforms for automotive telemetry applications [10,11,12,49,50].

Characteristic	GEO	MEO	LEO	HAPS
**Altitude**	≈35,786 km	≈8000–20,000 km	≈500–2000 km	≈20 km
**Round-trip latency**	≈480–560 ms	≈130–250 ms	≈30–50 ms	<10 ms
**Coverage per platform**	≈1/3 of Earth’s surface	Regional (thousands of km)	Footprint of hundreds of km	≈100–200 km diameter
**Platforms required for continuous service**	3–4	≈10–20	Hundreds to thousands	Local deployment per served area
**Handover frequency**	None (quasi-static)	Every few hours	Every ≈3–10 min	Rare (quasi-static)
**Doppler shift**	Negligible	Moderate	High (up to ≈±48 kHz at 2 GHz)	Negligible
**Typical telemetry role**	Delay-tolerant bulk reporting, broadcast updates	Navigation (GNSS), medium-latency backhaul	Near-real-time telemetry, IoT-NTN, emergency messaging	Temporary capacity extension, disaster recovery

**Table 3:** Comparison of communication technologies used in connected vehicle ecosystems.

Parameter	DSRC	C-V2X	5G	Satellite
**Coverage**	Local (≈300–1000 m)	Local–regional (≈1–2 km sidelink; wide-area via cellular Uu)	Regional (cell-based; dense deployment required at mmWave)	Global/near-global
**Latency**	Low (≈1–10 ms)	Low (≈10–50 ms; <20 ms NR sidelink)	Very low (1–10 ms, URLLC)	High (GEO ≈ 480–560 ms RTT; LEO ≈ 30–50 ms RTT)
**Data Rate**	Medium (3–27 Mbps)	High (up to ≈300 Mbps LTE; higher with 5G NR)	Very high (up to 10–20 Gbps peak)	Medium (≈10–100+ Mbps, LEO broadband)
**Mobility Support**	High	High	Very High	Medium
**Infrastructure Dependency**	Medium	Medium	High	Low
**Primary Applications**	Safety Messages	Cooperative Driving	Autonomous Mobility	Remote Connectivity
**Energy Efficiency**	High (low-power short-range radios)	Medium–High (sidelink power control)	Medium (dense infrastructure and mmWave increase energy cost)	Low–Medium (W-class terminal transmit power)
**Scalability**	Limited (channel congestion at high density)	High (network-scheduled resources, congestion control)	Very High (network slicing; up to 10^6^ devices/km^2^)	Medium (limited spectrum per beam)
**Deployment Cost and Complexity**	Low–Medium (mature, low-cost RSUs; shrinking ecosystem support)	Medium (reuses existing cellular infrastructure)	High (dense small cells, licensed spectrum)	High constellation CAPEX; minimal ground infrastructure
**Standardization Status**	IEEE 802.11p/bd; U.S. 5.9 GHz band largely reallocated	3GPP Rel-14–16 (LTE-V2X, NR sidelink)	3GPP Rel-15–18 (5G-Advanced)	3GPP NTN Rel-17–19 and proprietary systems

**Table 4:** Emerging technologies supporting intelligent connected mobility.

Technology	Main Function	Representative Applications	Indicative Quantitative Characteristics
**Artificial Intelligence**	Decision support and analytics	Autonomous driving	Onboard inference latency ≈ 1–50 ms on automotive SoCs; model sizes from MBs to GBs
**Digital Twins**	Virtual representation and simulation	Predictive maintenance	Synchronization intervals from ≈10 ms (control-level twins) to minutes (fleet-level twins)
**Edge Computing**	Low-latency processing	Real-time traffic management	End-to-end RTT typically <20 ms, vs. ≈50–200 ms for centralized cloud processing
**IoT**	Device connectivity	Vehicle monitoring	5G mMTC design target of up to 10^6^ connected devices per km^2^
**Blockchain**	Data integrity and trust	Secure data sharing	Typical permissioned-chain throughput ≈ 10^2^–10^4^ TPS; added confirmation latency ≈ 0.1–1 s
**Cloud Computing**	Large-scale data processing	Fleet management	Practically unlimited storage/compute; access latency ≈ 50–200 ms

**Table 5:** Cybersecurity threats and mitigation strategies in connected vehicle environments.

Threat Category	Potential Impact	Mitigation Strategy	Relevant Standards and Mechanisms
**Spoofing Attacks**	False information dissemination	Authentication protocols	IEEE 1609.2 [72] certificates; C-ITS credential management (PKI)
**Denial-of-Service Attacks**	Communication disruption	Network monitoring and filtering	ETSI decentralized congestion control; traffic filtering and rate limiting
**Malware Infections**	System compromise	Secure OTA updates	UNECE R156 software update management; secure boot
**Data Tampering**	Loss of data integrity	Blockchain verification	Hash chains; hardware security modules (HSM/TPM)
**Unauthorized Access**	Privacy violations	Encryption and access control	ISO/SAE 21434 access-control requirements; TLS 1.3
**Sensor Manipulation**	Incorrect decision-making	Intrusion detection systems	Multi-sensor plausibility checks; redundancy and voting schemes

**Table 6:** Prioritized research roadmap for long-range automotive telemetry in connected vehicles.

Research Gap	Priority	Indicative Horizon	Key Open Questions	Recommended Actions
**Seamless 5G–LEO service continuity**	High	Short term (1–3 years)	Session continuity at highway speeds; vertical handover latency	Fleet-scale trials with Release-17/18 NTN chipsets; standardized handover KPIs
**Common evaluation benchmarks**	High	Short term (1–3 years)	Comparability of latency, reliability, and scalability results	Open reference scenarios, shared datasets and testbeds
**Certifiable AI for telemetry analytics**	High	Medium term (3–5 years)	Explainability and verification under ISO 26262/SOTIF	Verification methods for deep models; safety cases for AI-based functions
**Techno-economic viability of hybrid networks**	High	Medium term (3–5 years)	Cost per vehicle; infrastructure business models	Public techno-economic models; regulator–operator–OEM cost-sharing studies
**Scalable digital twin synchronization**	Medium	Medium term (3–5 years)	Fidelity–latency trade-off; cross-vendor interoperability	Standardized twin interfaces; hierarchical synchronization architectures
**Privacy-preserving telemetry at scale**	Medium	Short–medium term (1–5 years)	Cross-border data flows; driver re-identification risk	Federated learning; standardized anonymization of mobility traces
**Post-quantum vehicular security**	Medium	Medium–long term (3–8 years)	Post-quantum cryptography within V2X latency budgets on constrained platforms	Lightweight PQC profiling; migration strategies aligned with ISO/SAE 21434

**Table 7:** Comparative overview of key technologies in connected vehicle ecosystems.

Technology	Main Advantages	Main Limitations	Key Applications	Future Research Challenges	Maturity (Indicative TRL)
**Automotive Telemetry**	Real-time data acquisition	Large data volumes	Monitoring, diagnostics	Data management	TRL 8–9 (commercially deployed)
**V2X Communication**	Extended situational awareness	Infrastructure dependency	Safety and traffic efficiency	Interoperability	TRL 6–8 (regional deployments ongoing)
**5G Networks**	Ultra-low latency	Coverage limitations	Autonomous driving	Network scalability	TRL 8–9 overall; URLLC features ≈ 5–7
**Satellite Communication**	Global coverage	Higher latency	Remote connectivity	Hybrid integration	TRL 4–6 for automotive integration
**Artificial Intelligence**	Intelligent decision-making	Computational requirements	Autonomous mobility	Explainability	TRL 5–8 depending on function
**Digital Twins**	Simulation and prediction	Model complexity	Optimization and maintenance	Real-time synchronization	TRL 4–6
**Blockchain**	Security and trust	Scalability issues	Secure data sharing	Computational efficiency	TRL 3–5 in vehicular context
**Edge Computing**	Low-latency processing	Resource constraints	Real-time services	Distributed orchestration	TRL 6–8

## Data Availability

Data are available upon request to the corresponding author.
